# Catastrophic health expenditure on private sector pharmaceuticals: a cross-sectional analysis from the state of Odisha, India

**DOI:** 10.1093/heapol/czac035

**Published:** 2022-04-27

**Authors:** Annie Haakenstad, Anuska Kalita, Bijetri Bose, Jan E Cooper, Winnie Yip

**Affiliations:** Hans Rosling Center for Population Health, Institute for Health Metrics and Evaluation, University of Washington, Guthrie Annex 3 (GA3), Seattle, WA 98121, USA; Department of Global Health and Population, Harvard T.H. Chan School of Public Health, 677 Huntington Avenue, Boston, MA 02115, USA; Department of Global Health and Population, Harvard T.H. Chan School of Public Health, 677 Huntington Avenue, Boston, MA 02115, USA; Department of Global Health and Population, Harvard T.H. Chan School of Public Health, 677 Huntington Avenue, Boston, MA 02115, USA; Department of Global Health and Population, Harvard T.H. Chan School of Public Health, 677 Huntington Avenue, Boston, MA 02115, USA; Department of Global Health and Population, Harvard T.H. Chan School of Public Health, 677 Huntington Avenue, Boston, MA 02115, USA

**Keywords:** Financial risk protection, catastrophic health expenditure, pharmaceuticals, drugs, out-of-pocket payments, India, universal health coverage

## Abstract

India has high rates of catastrophic health expenditure (CHE): 16% of Indian households incur CHE. To understand why CHE is so high, we conducted an in-depth analysis in the state of Odisha—a state with high rates of public sector facility use, reported eligibility for public insurance of 80%, and the provision of drugs for free in government-run facilities—yet with the second-highest rates of CHE across India (24%). We collected household data in 2019 representative of the state of Odisha and captured extensive information about healthcare seeking, including the facility type, its sector (private or public), how much was spent out-of-pocket, and where drugs were obtained. We employ Shapley decomposition to attribute variation in CHE and other financial hardship metrics to characteristics of healthcare, controlling for health and social determinants. We find that 36.3% (95% uncertainty interval: 32.7–40.1) of explained variation in CHE is attributed to whether a private sector pharmacy was used and the number of drugs obtained. Of all outpatient visits, 13% are with a private sector chemist, a similar rate as public primary providers (15%). Insurance was used in just 6% of hospitalizations and its use explained just 0.2% (0.1–0.4) of CHE overall. Eighty-six percent of users of outpatient care obtained drugs from the private sector. We estimate that eliminating spending on private drugs would reduce CHE by 56% in Odisha. The private sector for pharmaceuticals fulfills an essential health system function in Odisha—supplying drugs to the vast majority of patients. To improve financial risk protection in Odisha, the role currently fulfilled by private sector pharmacies must be considered alongside existing shortcomings in the public sector provision of drugs and the lack of outpatient care and drug coverage in public insurance programs.

Key messages

- 86% of drug purchases occur in the private sector and 2.5 drugs are obtained per outpatient visit, contributing to 36.3% (32.7–40.1) of explained variation in catastrophic health expenditure (CHE).
- The number of CHE cases would be reduced by 56% if all private drug purchases were substituted with free public drugs or covered by insurance programs or government subsidies.
- Private sector chemist shops play a more substantial role in outpatient care than previously thought, serving as the site of 13% of outpatient visits—more than AYUSH providers (6%) and private primary care providers (4%), and similar in share to public primary facilities (15%).
- Existing health insurance schemes that cover hospitalization costs explained 0.2% (0.1–0.4) of CHE, providing limited financial risk protection due to low awareness of eligibility, low uptake when hospitalized and lack of coverage of outpatient care and drugs obtained outside the facility where hospitalized.

## Introduction

In India, a major portion of households faces financial hardship due to healthcare costs, threatening the health and prosperity of Indians and the country’s pursuit of universal health coverage. In 2017/2018, 16% of households incurred catastrophic health expenditure (CHE) or when healthcare costs exceed 10% or more of household consumption expenditure (National Sample Survey Office, 2019). India performs poorly relative to its peers: in other countries in the World Bank’s lower-middle-income group, just 9% of households faced CHE (The World Bank, 2019).

India has strived to address poor financial risk protection through various public policies, including by providing health services and drugs free of charge in the public sector (Bajpai *et al.*, 2009). While an effective approach in theory, these programs are underused in practice, in part because the government invests less than peer countries in health. The Indian government spent just 0.9% of GDP on health compared to 2.4% in other lower-middle-income countries (Institute for Health Metrics and Evaluation (IHME), 2020). Faced with poorly funded public health facilities, many Indian patients pursue care in the private sector instead—47% of all hospitalizations and 58% of all outpatient visits in 2017/2018 across India were in the private sector (National Sample Survey Office, 2019). In 2018, out-of-pocket (OOP) was 23% of total health expenditure in India, amounting to $45 per person (Institute for Health Metrics and Evaluation (IHME), 2020).

Government-run insurance programs, Pradhan Mantri Jan Arogya Yojana, its predecessor Rashtriya Swasthya Bima Yojana (RSBY), and state-run programs also aim to improve financial protection by covering the costs of hospitalizations in public and private facilities (Official Website Ayushman Bharat; Rashtriya Swasthya Bima Yojana (RSBY); Devadasan *et al.*, 2013; Ghosh, 2014). Quasi-experimental studies have found that RSBY and insurance programs in Andhra Pradesh, Tamil Nadu and Karnataka resulted in lower OOP spending and borrowing but did not reduce CHE or impoverishing health expenditures (Katyal *et al.*, 2015; Rao *et al.*, 2014; Sood *et al.*, 2014; Bergkvist *et al.*, 2014; Fan *et al.*, 2012; Karan *et al.*, 2017; Dhanaraj, 2014). Evidence from observational studies and research on conditional cash transfers and community-based health insurance also show that reducing OOP spending on hospitalizations has not been effective in improving financial protection (Tripathi *et al.*, 2014; Mukherjee and Singh, 2018; Aggarwal, 2010; Devadasan *et al.*, 2007; Ranson, 2002).

Instead, drugs and outpatient care seem to be key drivers of CHE. An estimated 56% of OOP costs and 67% of all CHE cases across India are due to spending on drugs alone (National Sample Survey Office, 2019). However, key questions remain about why outpatient care and drugs are such major contributors to CHE, given that the public sector provides drugs for free and India is the second-biggest producer of drugs by volume worldwide (McKinsey & Company, 2021). A key gap in the evidence base is the role of the private sector for drugs. Large household surveys in India do not capture consultations with pharmacists nor whether drugs are purchased in the private sector (National Sample Survey Office, 2019). Among 123 peer-reviewed studies on CHE in India (Supplementary Appendix Table A1), just two had any analysis of the private sector for drugs, and these analyses were limited to a single row in a table or a single disease area (Gwatidzo and Stewart Williams, 2017; Gupt *et al.*, 2016). No existing studies investigate in-depth the role of private sector chemists in driving up the OOP costs of outpatient care and drugs.

In this study, we assess the causes of high CHE in the Empowered Action Group (EAG) state of Odisha, where OOP is 76% of total health expenditure (Rout *et al.*, 2016) and CHE rates are the second highest (24%) across Indian states (National Sample Survey Office, 2019). While OOP spending per person in Odisha is 16th highest across Indian states, consumption expenditure is lowest nationwide—both high OOP and low consumption expenditure contribute to high rates of CHE in Odisha (National Sample Survey Office, 2019). OOP is relatively high despite the majority of Odisha’s households seeking care at public sector facilities. Additionally, 80% of households in Odisha are eligible for the state-run insurance program Biju Swasthya Kalyan Yojana (BSKY), which, like other programs, covers hospitalizations but not outpatient care or drugs. Recognizing the high OOP costs of drugs, Odisha launched the Niramaya program in 2015 to strengthen drug supply in the public sector and improve access to free, public drugs (Department of Health and Family Welfare, 2021).

Given the state’s efforts to provide insurance coverage to a large share of the population and invest in the public provision of drugs, we set out to understand why OOP and CHE remain persistent problems in Odisha. We collected extensive data from households about their use of healthcare and the associated OOP costs, including, for the first time, how frequently private sector chemists are patients’ first contact with the health system, despite pharmacists being prohibited from providing medical advice in India. Our primary interest is to attribute poor financial risk protection to ‘healthcare’ determinants amenable to policy change, controlling for health and social determinants. We argue that, in order to improve financial risk protection, Odisha must grapple with the essential function played by the private market for pharmaceuticals.

## Methodology

### Data

We collected data from 7567 households and 30 645 individuals in 2019 in Odisha, India. A multi-stage clustered sampling design was used to select households to participate in the survey and ensure a state-representative sample (details in Supplementary Appendix, pages 19–24). We determined sample size based on requiring a sufficient number of hospitalized patients. Sampling weights were developed based on the probability of selection, and iterative proportionate raking was used to calibrate the weights to known population totals for the state of Odisha (Supplementary Appendix, pages 24–26). We validated the representativeness of our household survey with the 2017/2018 National Sample Survey (NSS) (Supplementary Appendix, pages 27–30). Institutional Review Board (IRB) approval for this study was obtained from the authors’ institution, an independent IRB, SIGMA, in India to meet domestic requirements, and the health research approval committee of the Government of Odisha.

Our household survey captured OOP health care costs from three different perspectives. First, respondents were asked to report total OOP expenditure for the household for doctor fees, diagnostic tests, drugs, and other healthcare costs for the 30 days prior to the survey and hospitalizations for the year prior to the survey. Second, the survey asked about details for each outpatient visit in the household in the 15 days prior to the survey, including where care was sought, how much was spent for each visit and what spending purchased. While national surveys like the NSS lump pharmacists into an ‘other’ health provider category, we explicitly asked whether a respondent visited a pharmacy when ill and considered the visit to be an outpatient visit if they asked the pharmacist for advice for their medical condition. Visits in which drugs were obtained only were not considered to be an outpatient visit. Finally, the survey asked about the OOP costs of every hospitalization in the year prior to the survey, the location of care, and other characteristics of hospitalizations. Reimbursements were removed from all OOP costs. We use the average 2019 exchange rate of 0.014 dollars to a rupee to convert OOP to US dollars.

We examine financial risk protection with four metrics. First, we depict log OOP costs per outpatient and inpatient visit (with an offset of 10% of the median added to address the presence of zeroes). Second, we use OOP as a share of consumption expenditure (OOP/CE), transformed with an offset (10% of the median) and a natural log. We include zeroes in all OOP expenditure values. Consumption expenditure was constructed based on summing reported household expenditure on housing, education, rent, food, and other areas. Different recall periods were made consistent over time by scaling expenditure to the same time frame for all categories (e.g. multiplying spending for a 7-day period by 4.3 to match a 30-day recall period). We use the continuous, underlying construct for CHE measures extensively to depict spending and utilization because OOP/CE represents variation in financial hardship due to healthcare costs better than the binary CHE measure, which simply indicates the passing of a threshold. Where OOP expenditure was not undertaken, it is represented by zero in the OOP/CE variable. Third, we construct CHE based on OOP surpassing 10% or 25% of consumption expenditure, as in Sustainable Development Goal target 3.8 2 (World Health Organization (WHO), 2021). The final financial risk protection metric is distress financing—whether borrowing or the selling of assets were required to cover OOP costs, a more severe form of financial hardship than CHE.

### Decomposition analysis

We employ Shapley decomposition to quantify how much determinants explain variation in our four financial hardship metrics (CHE, OOP/CE, OOP and distress financing) and whether there was any OOP spending (Lindeman *et al.*, 1980; Gromping, 2006). We adopt this approach because, in a basic regression analysis, the coefficient values do not quantify how much each factor explains variation in the outcome. While an association could be large, the actual change in the factor may be relatively small in magnitude and thus not explain much of the variation in the outcome. Therefore, Shapley decomposition attributes the explained variation (*R*^2^) in the outcome into contributions from each of the factors in the analysis. The approach involves averaging over orders as in Lindeman, Merenda, and Gold (1980). Conceptually, we run a regression for each combination of covariates. A given covariate’s contribution to variation is based on the average change in the *R*^2^ when the covariate is added to the regression across all combinations of covariates. One thousand bootstrap draws were used to quantify uncertainty.

Our econometric model is as follows:

$$Y &= \beta *healthcare{\ }determinants + \gamma *health{\ }determinants \\ &\quad+ \delta *social{\ }determinants + \varepsilon $$

**Figure 1.**
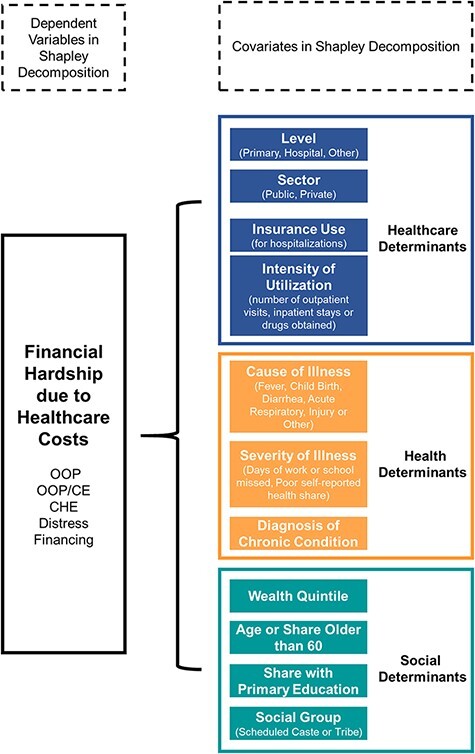
Determinants of financial hardship due to healthcare costs

Notes: OOP: out-of-pocket spending; OOP/CE: out-of-pocket spending as a share of consumption expenditure; CHE: catastrophic health expenditure—when OOP exceeds 10% as a share of consumption expenditure; distress financing: selling assets or borrowing required to cover OOP costs.

Here $Y$ represents our outcomes: whether or not a household had any OOP spending, natural log OOP spending per outpatient visit, natural log OOP spending per hospitalization, CHE at the 10% threshold, OOP as a share of consumption expenditure (OOP/CE) and whether borrowing or asset selling was required to cover costs (distress financing). Regressions were run with ordinary least squares with clustered standard errors. A linear probability model was used for all binary outcomes (whether or not OOP spending occurred, CHE, and distress financing) and other variables bounded between 0 and 1 (OOP/CE). We attribute variation in financial hardship due to OOP costs according to healthcare, health, and social determinants (Figure 1), grouping determinants according to whether they are directly addressable with health system reform (healthcare) or need further investigation to show how they can be modified with health policy (health, social). The coefficients in our econometric model represent a vector for each of the variables included in each determinant group ($\beta $, $ \gamma ,$$\delta $). Healthcare determinants capture characteristics of health service use as self-reported by individuals in our household survey: the volume or intensity of healthcare use (the number of outpatient visits in the 15 days prior to the survey, natural log of inpatient stays in the year prior to the survey and the number of drugs obtained for outpatient care in the 15 days prior to the survey), sector (private versus public), level (primary, hospital, chemist or other non-provider care), and the use of insurance (for hospitalizations). Coding of health facilities into sectors and levels is presented in the Supplementary Appendix (Table A.12). In our data, the use of the private sector for obtaining drugs is only available for outpatient care.

We also examine health and social determinants to rule out alternative drivers of financial hardship. Health determinants in our analysis pertain to the severity and type of illness. We used the following health determinants as self-reported by respondents: whether individuals reported any diagnosis by a healthcare provider of a chronic condition: whether individuals reported that, in general, for their age, their health was poor: the number of days of school or work reported as missed due to illness in the last 15 days; and which disease or condition was reported to have afflicted the individual among the most frequently reported types of illness (fever, childbirth, injury, diarrhoea and acute respiratory infection) (details in Supplementary Appendix, Table A.13). Finally, social determinants in our analysis pertain to characteristics outside of health and healthcare that affect households’ engagement with the health system, factors that increase susceptibility to disease, and factors that may affect whether household budgets can absorb large healthcare costs. We include the following: asset-based wealth quintile; rural/urban residence as determined by the categorization of the location in the census; education (the share with only primary education in the household or whether primary education was obtained among outpatients and inpatients), age (whether anyone was older than 60 years in a household or the age of outpatients and inpatients) and social group (belonging to scheduled tribe or scheduled caste groups).

Analysis was conducted in Stata 14.0 and R 4.0.3.

## Results

### Characteristics of households and individuals

The household survey captured a sample representative of the state of Odisha. Table 1 provides information on basic demographics and expenditure characteristics of the individuals and households surveyed. We surveyed 7567 households and 30 645 individuals, most residing in rural areas (84%) and around one-fifth self-identifying as belonging to either scheduled tribe (22%) or scheduled caste (17%) groups, respectively. CHE was incurred by 24% of households, the same rates as reported in the most recent NSS (Supplementary Appendix, Table A.7). Among all CHE cases, 65% could be attributed solely to drug spending.

**Table 1.** Characteristics of the household survey sample

	Value	Standard error (Min, Max)
Household and individual demographic characteristics		
Number of households	7567	
Number of individuals	30 645	
Share scheduled tribe	22%	2.4
Share scheduled caste	17%	1.2
Share rural	84%	2.6
Share female	50%	0.4
Share married	56%	0.4
Share primary school or fewer years of education	9%	0.3
Share under 5	8%	0.3
Share under 18	31%	0.5
Share over 60	11%	0.4
Report any insurance	14%	0.9
Share with chronic diagnosis	8%	0.3
Share with poor self-reported health	1%	0.1
Household consumption & health expenditure		
Average annual consumption expenditure	$1708	68.7 (42.1, 126915.9)
Median annual consumption expenditure	$1199	
Average health expenditure	$161	8.0 (0, 85 806)
Median annual out-of-pocket health expenditure	$4	
Average out-of-pocket health expenditure share of consumption expenditure	8%	0.2 (0, 99.3)
Share of households with any out-of-pocket health spending	43%	3.9
CHE		
Share with CHE at 10%	24%	0.8
Share with CHE at 25%	10%	0.6
Share of CHE at 10% due to drugs	65%	1.6
Share of CHE at 10% due to hospitalization	22%	1.5
Distress financing	8%	0.4
Care-seeking		
Share ailing in last 15 days	11%	0.3
Days of school work missed in last 15 days due to ailment	2.6	0.1 (0,15)
Share of ailing in the last 15 days that sought treatment	91%	0.8
Share that did not seek care because not sick enough	85%	3.0
Share that did not seek care because of costs	9%	2.3
Share of individuals using outpatient care in last 15 days	11%	0.3
Private sector share of outpatient visits in the last 15 days (including private chemists, private Ayush and private other non-providers)	48%	1.8
Wait time (minutes)	24.8	1.2 (0,576)
Fever share of outpatient visits	57%	1.7
Diarrhoea share of outpatient visits	3%	0.5
Child Birth share of outpatient visits	2%	0.3
Injury share of outpatient visits	2%	0.4
Acute Respiratory share of outpatient visits	8%	0.7
Share of individuals with hospitalizations in the last 365 days	4%	0.1
Private sector share of hospitalizations in the last year	25%	1.6
Fever share of hospitalizations	16%	1.3
Diarrhoea share of hospitalizations	5%	1.2
Child Birth share of hospitalizations	28%	1.6
Injury share of hospitalizations	6%	0.6
Acute Respiratory share of hospitalizations	2%	0.5

Notes: CHE calculated based on whether household out-of-pocket healthcare costs exceeded 10% or 25% of consumption expenditure.

Among individuals surveyed, 11% were ailing in the last 15 days and 91% of ailing individuals sought treatment. Among the 9% of ailing individuals that did not seek care, 85% reported not being sick enough to warrant care-seeking. Across individuals surveyed, 11% used outpatient care in the 15 days prior to the survey. Visits to the private sector comprised 48% of outpatient visits—this higher amount than the NSS is explained by the inclusion of chemists, AYUSH practitioners, and other providers in the private sector. Hospitalization rates were 4% in the year prior to the survey, with 25% of inpatient stays occurring in private sector facilities.

### Characteristics of healthcare

In Table 2, we break down outpatient visits and inpatient stays by where care was sought. Of all outpatient care in the last 15 days, 13% of visits were to private sector chemists. We distinguished a visit at which a chemist was asked advice (counted as an outpatient visit) versus a situation where only drugs were purchased (excluded from the outpatient visit count). On average, patients that received outpatient care spent a total of $11.9 per visit and 26% of all outpatient visits entailed CHE. A substantial share of OOP costs for outpatient care went to drugs: $7.4 per visit or 57% of total OOP outpatient care costs went to drugs. Furthermore, 86% of outpatients purchased at least one drug from a private sector pharmacy. Even in public sector hospitals and public primary facilities, where drugs are supposed to be provided free of charge, more than 70% of patients reported purchasing drugs from the private sector; drugs are 50% and 54% of all costs in these facilities.

**Table 2.** Characteristics of outpatient care and hospitalizations

Outpatient care in the last 15 days										
	**Share of outpatient visits**	**Share of visits with any OOP costs** **(SE)**	**Mean total OOP cost per visit, USD** **(SE, Min, Max)**	**Mean Drug OOP cost per visit, USD** **(SE, Min, Max)**	**Drugs share of OOP** **(SE)**	**Mean number of drugs** **(SE, Min, Max)**	**Share of visits where drugs obtained from private sector** **(SE)**	**Share of visits resulting in CHE (10%) (monthly CE)** **(SE)**	**Share of outpatient CHE (10%) cases** **(SE)**	**Share of visits resulting in distress financing** **(SE)**
Public hospital	27%	90% (1)	$13.0 (1.0, 0.0, 602.0)	$6.3 (0.4, 0.0, 140.0)	50% (2)	3.0 (0.1, 0.0, 35.0)	71% (3)	30% (2)	34%	11% (2)
Public primary	15%	79% (3)	$7.3 (0.7, 0.0, 168.0)	$4.9 (0.5, 0.0, 140.0)	54% (3)	2.7 (0.1, 0.0, 30.0)	75% (3)	15% (2)	10%	10% (2)
Private hospital	20%	97% (1)	$22.7 (2.1, 0.0, 1,169.0)	$11.8 (1.1, 0.0, 770.0)	61% (2)	3.1 (0.1, 0.0, 12.0)	100% (0)	41% (3)	31%	17% (2)
Private primary	4%	98% (1)	$11.6 (3.6, 0.0, 378.0)	$8.2 (2.6, 0.0, 252.0)	79% (4)	1.9 (0.3, 0.0, 10.0)	100% (0)	30% (8)	3%	11% (4)
Private chemist	13%	98% (1)	$7.8 (1.2, 0.0, 490.0)	$5.4 (0.5, 0.0, 98.0)	78% (2)	2.0 (0.2, 0.0, 15.0)	100% (0)	24% (4)	9%	5% (1)
Ayush^a^	6%	98% (1)	$18.1 (3.1, 0.0, 273.0)	$10.3 (1.0, 0.0, 105.0)	66% (4)	2.8 (0.2, 0.0, 8.0)	100% (0)	38% (5)	9%	7% (2)
Other non-provider^b^	16%	52% (4)	$3.5 (0.6, 0.0, 112.0)	$5.5 (1.0, 0.0, 56.0)	41% (4)	1.1 (0.1, 0.0, 10.0)	96% (2)	8% (2)	4%	2% (1)
Total	100%	86%	$11.9	$7.4	57%	2.5	86%	26%	100%	10%
		(1)	(0.6, 0.0, 1,169.0)	(0.3, 0.0, 770.0)	(1)	(0.1, 0.0, 35.0)	(1)	(1)		(1)
**Hospitalizations in the last year**										
	**Share of hospitalizations**	**Share of hospitalizations with any OOP costs**	**Mean Total OOP cost per visit, USD** **(SE, Min, Max)**	**Mean drug OOP cost per visit, USD** **(SE, Min, Max)**	**Drugs share of OOP**	**Report use insurance for hospitalization**	**Reimbursed by insurance for hospitalization**	**Share of hospitalizations resulting in CHE (10%) (Yearly CE)**	**Share of hospitalization CHE (10%) Cases**	**Share of hospitalizations resulting in distress financing**
Public hospital	75%	96% (1)	$146.1 (11.9, 0.0, 11,200.0)	$49.3 (4.5, 0.0, 4,200.0)	42% (2)	5% (1)	4% (1)	19% (2)	53%	38% (2)
Private hospital	25%	94% (2)	$477.4 (37.9, 0.0, 11,200.0)	$107.3 (18.7, 0.0, 2,649.8)	27% (2)	9% (2)	8% (2)	52% (4)	47%	45% (4)
Total	100%	95%	$223.8	$62.9	38%	6%	5%	27%	100%	40%
		(1)	(14.7, 0.0, 11,200.0)	(6.1, 0.0, 4,200.0)	(1)	(1)	(1)	(2)	–	(2)

Notes: ^a^AYUSH and other non-provider include both private and public providers. ^b^ Other non-provider includes traditional healer; ‘Bengali doctor’, or other names; and other stores (e.g. grocery stores). OOP: out-of-pocket spending; CHE: catastrophic health expenditure measured as OOP as 10% or more of consumption expenditure. For outpatient CHE cases, a monthly consumption expenditure amount was used, whereas for hospitalization CHE cases, the consumption expenditure denominator was for the year.

Table 2 also depicts characteristics of hospitalizations in the last year. The private sector plays a smaller role in the share of hospitalizations (25%) but is more expensive overall. The total share of drugs in hospitalization OOP is 38%. Just 5% of patients hospitalized in the public sector and 9% of patients hospitalized in the private sector reported using insurance for their visit.

**Figure 2.**
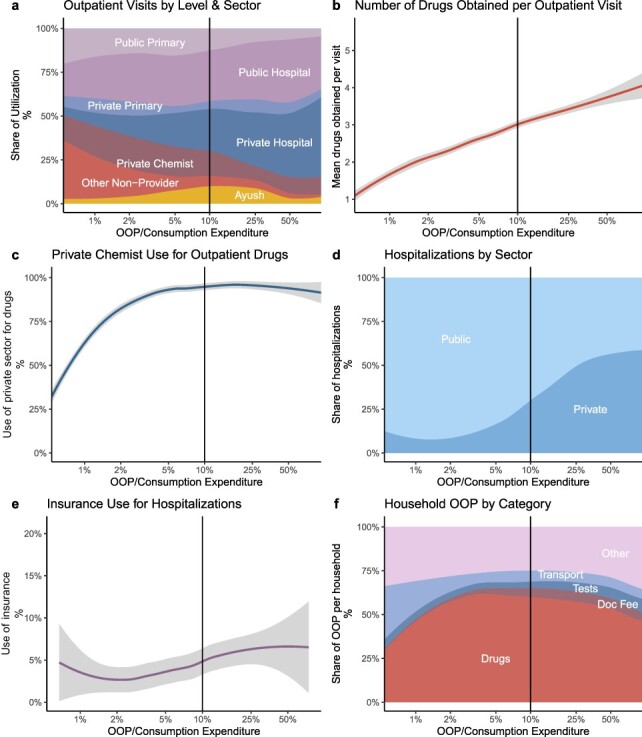
Characteristics of outpatient visits, hospitalizations and OOP costs

Notes: OOP: out-of-pocket spending. Smoothing of plot inputs estimated through loess regression.

Figure 2 depicts characteristics of outpatient care, hospitalizations and total household OOP costs against the OOP share of consumption expenditure. The black vertical line in each figure represents the 10% consumption expenditure threshold after which health care spending is considered catastrophic. Figure 2a shows that as OOP/consumption expenditure rises, private sector hospitals grow as a share of all outpatient visits and the other non-provider category declines. The share of public sector providers is steady across OOP/consumption expenditure levels, although public primary care declines and public hospital use increases as spending increases. Figure 2b and c shows that the number of drugs obtained by the patients rises as spending as a share of consumption expenditure increases, and the share of drugs obtained in the private sector asymptotes close to 100% before the 10% CHE threshold. Figure 2d shows that as the OOP share of consumption expenditure rises, the private sector share of hospitalizations also increases. Figure 2e shows little variation in the reported use of insurance as OOP as a share of consumption expenditure changes. Finally, Figure 2f presents the breakdown of spending by cost category, showing that drug costs comprise more than 50% of OOP even as spending extends beyond the 10% CHE threshold.

### What explains variation in health expenditure?

**Figure 3.**
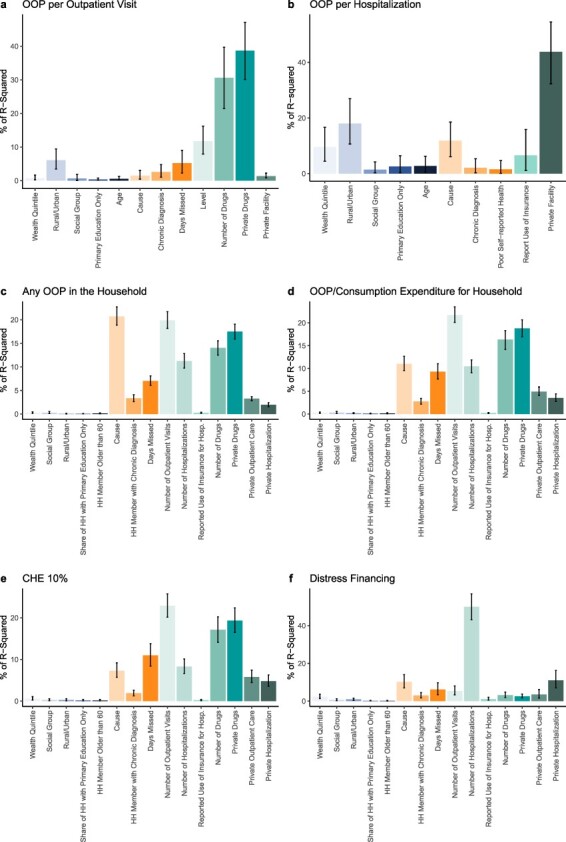
Shapley decomposition results

Notes: OOP: out-of-pocket spending; OOP/CE: out-of-pocket spending as a share of consumption expenditure; CHE: catastrophic health expenditure—when OOP exceeds 10% as a share of consumption expenditure; distress financing: selling assets or borrowing required to cover OOP costs. The share of *R*^2^ estimated through Shapley decomposition of the linear regressions reported in Table 3. OOP per outpatient visit and per hospitalization as well as OOP/consumption expenditure for household natural log-transformed.

Figure 3 depicts the results of the decomposition analysis and Table 3 reports the regression coefficients from the underlying linear models (details in Supplementary Appendix Table A.14). For the financial hardship measures captured at the household level (OOP/CE, CHE 10% and distress financing), healthcare determinants are associated with 76.0–78.3% of all the explained variation. In contrast, health determinants explained between 19.3 and 23.1% and social determinants were attributed 4.0% or less of explained variation. The largest healthcare determinants of OOP/CE (Figure 3d) were the number of outpatient visits (21.7%, 95% uncertainty interval: 20.5–22.8), whether drugs were obtained in the private sector (18.9%, 17.6–20.5), the number of drugs obtained (16.4%, 14.6–18.0), the cause of visit (11.0%, 9.6–12.5) and the days missed (9.3%, 8.3–10.7). All other determinants were associated with less than 5% of explained variation. Similarly, the combination of obtaining drugs in the private sector and the number of drugs obtained contributed substantially to the explained variation in OOP costs per outpatient visit (69.3%, 61.3–76.2), any OOP in the household (31.4%, 29.6–33.2), OOP/CE (35.3%, 32.5–37.5) and CHE 10% (36.3%, 32.7–40.1).

As shown in Table 3, for each outpatient care visit, OOP/CE increased 0.73 on the log scale or 7.6 percent (*P* < 0.001); for each additional drug obtained, OOP/CE increased 3 percent (*P* < 0.001). If drugs were obtained in the private sector, OOP/CE increased by 23 percent (*P* < 0.001). Distress financing was unique in that just one covariate—the number of hospitalizations—was associated with 50.1% (45.2–54.9) of all explained variation—each hospitalization was associated with a 26 percentage point increase in distress financing (*P* < 0.001). Finally, for hospitalizations (Figure 3b) for which detailed information on drugs was not obtained, using care in the private sector was associated with 43.9% (34.5–52.5) of the explained variation and an 1.10 increase in log OOP per hospitalization (*P* < 0.001) or 3 times higher OOP costs than public sector hospitalizations. The reported use of insurance to cover the costs of hospitalization is associated with just 6.8% (1.5–13.8) of explained variation, although it is a statistically significant negative predictor of OOP/CE (−0.93, *P* < 0.001), representing a 40% decline in OOP per hospitalization when insurance was reportedly used, controlling for other covariates.

## Discussion

**Table 3.** Regression results

	Dependent variable:					
	Log OOP per outpatient visit	Log OOP per hospitalization	Any OOP	Log OOP/CE	CHE 10%	Distress financing
	(1)	(2)	(3)	(4)	(5)	(6)
Rural	0.166^**^	0.258^**^	0.022^**^	0.135^***^	0.026^**^	0.028^***^
	(0.076)	(0.112)	(0.011)	(0.034)	(0.013)	(0.007)
Wealth quintile 1 (reference)	–	–	–	–	–	–
Wealth quintile 2	−0.011	0.042	0.003	−0.127^***^	−0.032^**^	0.002
	(0.097)	(0.125)	(0.015)	(0.049)	(0.015)	(0.010)
Wealth quintile 3	−0.018	0.367^***^	−0.010	−0.106*	−0.020	−0.001
	(0.095)	(0.118)	(0.015)	(0.057)	(0.016)	(0.012)
Wealth quintile 4	0.005	0.259^**^	−0.003	−0.134^**^	−0.015	−0.034^***^
	(0.096)	(0.130)	(0.014)	(0.056)	(0.019)	(0.013)
Wealth quintile 5	0.008	0.183	0.007	−0.245^***^	−0.065^***^	−0.073^***^
	(0.106)	(0.127)	(0.016)	(0.062)	(0.021)	(0.013)
Other social group (reference)	–	–	–	–	–	–
Scheduled caste	−0.066	−0.172	0.003	−0.018	−0.001	−0.017
	(0.092)	(0.112)	(0.010)	(0.043)	(0.013)	(0.012)
Scheduled tribe	−0.125	−0.617^***^	−0.011	−0.093*	−0.014	−0.030^***^
	(0.081)	(0.110)	(0.013)	(0.048)	(0.013)	(0.010)
Share with primary school or less	0.156^**^(0.075)	−0.192 (0.204)	0.021 (0.013)	0.124^**^ (0.051)	0.036^**^ (0.018)	−0.009 (0.013)
Age	−0.0001	0.003				
	(0.001)	(0.002)				
Chronic diagnosis	0.166^**^	0.122	0.085^***^	0.227^***^	0.020	0.028^**^
	(0.074)	(0.097)	(0.012)	(0.040)	(0.013)	(0.011)
Any HH member older than 60			−0.005 (0.007)	0.013 (0.029)	0.007 (0.010)	−0.012 (0.009)
Days of school of work missed in last 15 days due to illness	0.051^***^ (0.009)		0.017^***^ (0.004)	0.107^***^ (0.015)	0.028^***^ (0.005)	0.014^***^ (0.003)
Poor self-reported health		0.206				
		(0.150)				
Report use insurance for hospitalization		−0.930^***^ (0.256)	−0.087 (0.069)	−0.480^**^ (0.242)	−0.120 (0.086)	−0.081 (0.070)
Reason: acute respiratory (reference)	–	–	–	–	–	–
Reason: child birth	0.677^***^	0.023	0.086^**^	0.404^***^	0.134^***^	0.011
	(0.200)	(0.282)	(0.041)	(0.133)	(0.050)	(0.038)
Reason: diarrhoea	0.037	−0.634^**^	−0.040	−0.146	0.004	0.070
	(0.153)	(0.295)	(0.056)	(0.157)	(0.059)	(0.053)
Reason: fever	0.056	−0.599^**^	0.108^***^	0.363^***^	0.084*	−0.008
	(0.085)	(0.297)	(0.033)	(0.128)	(0.048)	(0.025)
Reason: injury	0.350	0.401	0.011	0.233	0.108	0.129^**^
	(0.246)	(0.322)	(0.051)	(0.192)	(0.069)	(0.060)
Reason: other or none	0.566^***^	0.007	−0.235^***^	−0.194*	0.061	0.015
	(0.095)	(0.287)	(0.030)	(0.105)	(0.040)	(0.025)
Number of outpatient visits in the last 15 days			0.194^***^ (0.018)	0.734^***^ (0.052)	0.156^***^ (0.015)	0.025^**^ (0.012)
Number of hospitalizations in the last year			0.266^***^ (0.023)	0.926^***^ (0.070)	0.154^***^ (0.017)	0.260^***^ (0.022)
Number of drugs obtained for outpatient visit	0.251^***^ (0.039)		0.020^***^ (0.007)	0.139^***^ (0.033)	0.034^***^ (0.010)	0.003 (0.005)
Outpatient drugs obtained in private sector	1.389^***^ (0.104)		0.267^***^ (0.032)	0.991^***^ (0.127)	0.205^***^ (0.037)	0.009 (0.024)
Level: hospital (reference)	–					
Level: other	−0.272^***^					
	(0.071)					
Level: primary	−0.402^***^					
	(0.085)					
Private outpatient care	−0.067		−0.033*	0.132*	0.045^**^	0.045^**^
	(0.059)		(0.019)	(0.078)	(0.019)	(0.018)
Private inpatient care		1.101^***^	0.084^***^	0.826^***^	0.245^***^	0.096^**^
		(0.101)	(0.029)	(0.115)	(0.030)	(0.041)
Constant	3.851^***^	8.407^***^	0.269^***^	−5.009^***^	−0.056	0.001
	(0.181)	(0.306)	(0.038)	(0.128)	(0.045)	(0.029)

Note: *P < 0.1; **P < 0.05; ***P < 0.01.

Our analysis shows that use of the private sector, including the private market for drugs, is an important determinant of poor financial risk protection in Odisha. In our decomposition analysis, the combination of the number of drugs obtained and private sector drug purchases was associated with more than 30% of explained variation in OOP costs per outpatient visit, OOP/CE, and CHE (Figure 3). Drugs comprised 57% of all OOP costs of outpatient care and 38% of hospitalization OOP costs. Hospitalization in a private facility cost more than three times as much as a public hospitalization (*P* < 0.001, Table 3) and was associated with more than 40% of the explained variation in OOP costs (Figure 3). Thus, to improve financial risk protection in Odisha, policymakers must consider the essential health system function played by the private sector, including the private provision of drugs.

In an EAG state with high rates of public sector use, private sector chemists play an essential role in supplying pharmaceuticals. 86% of all outpatient visits involved obtaining drugs from the private sector. Assuming the rate of private sector chemist use applies to both outpatient and inpatient care, CHE in the state would be reduced by 56% by eliminating spending at private sector pharmacies.^1^

Three factors explain the use of the private sector for drugs when drugs are supposed to be provided free of charge in the public sector. First, patients may be forced to use the private sector if needed drugs are out-of-stock at public facilities (Prinja *et al.*, 2013). In facility and chemist surveys conducted at the same time as our household survey, we found private chemists had more essential medicine list drugs in stocks than public primary facilities but fewer than public hospitals and community health centres (Supplementary Appendix, Table A.15). Second, providers may prescribe drugs that are only stocked in the private sector (Kotwani *et al.*, 2007). Providers may gain financially from prescribing certain drugs or sending patients to private chemist shops—for instance, through kickbacks from drug companies or financial interest in private chemist shops. Third, patients may ‘bypass’ stocked public sector drugs because they prefer the branded and branded generic drugs stocked at private chemists (Kotwani *et al.*, 2007). The existing literature suggests Indians perceive branded and branded generic drugs stocked at private pharmacies to be of higher quality than those available at public sector facilities, which tend to be generic drugs (Bhargava and Kalantri, 2013; Shahrawat and Rao, 2012; Tripathi and Bhattacharya, 2018; Aivalli *et al.*, 2018; Sreenivasan and Narasimha, 2018)^.^

Our analysis also revealed that private chemists and private sector providers, in general, played an important role in outpatient care. Surprisingly, 13% of outpatient visits were with private sector chemists, nearly as much as public primary facilities (15%). It has been noted that pharmacists are the first point of contact in a number of studies in low- and middle-income countries (Sudhinaraset *et al.*, 2013). The role of chemists as the first contact in India in particular is an important consideration as the country strives to bolster primary care—why do some patients seek advice from chemists rather than from government-run primary care facilities? Important potential factors include the convenience of longer opening hours of chemists (Supplementary Appendix Table A.17) or low perceived quality of care at primary public facilities. However, chemists are not qualified to diagnose and treat illnesses, raising serious questions about the quality of care for minor illnesses, some of which may develop into more serious cases. If chemists are generous with the provision of antibiotics in these contacts, the use of chemists for outpatient care has the potential to fuel antimicrobial resistance in India (Kumar *et al.*, 2013).

Insurance contributed little to no protection from financial risks. Reported use of insurance was a statistically significant, negative predictor of OOP costs per hospitalization (*P* < 0.001) and OOP/CE (p.001) (Table 3), but just 6% of patients reported using insurance for their hospital stay and insurance explained less than 7% of variation in all decomposition models. Only 14% of households reported having insurance (Table 1), despite state projections that 80% of households are eligible (Rout *et al.*, 2016). Low rates of reported eligibility may be related to adverse selection—households may only enrol once they are in need of insurance coverage for hospitalization, a relatively rare event. However, this does not explain low rates of insurance use for hospitalizations. The challenge for Odisha is to ensure people are aware they are covered by BSKY, use it for inpatient care, and that the programme actually protects patients from financial hardship.

We note that the number of outpatient and inpatient visits combined explained more than 30% of OOP incidence at the household level, OOP/CE, and CHE; the number of hospitalizations alone explained 50.1% (45.2–54.9) of all distress financing. This indicates demand is a key determinant—patient choices about whether to use care are critical to the incidence of financial hardship. However, utilization rates are not significantly higher in Odisha than in other Indian states (National Sample Survey Office, 2019).

An array of policy considerations pertain to improving financial risk protection in Odisha (Roberts *et al.*, 2008). First, improving awareness among beneficiaries of eligibility for BSKY and its coverage of hospitalization costs is critical to ensuring the insurance programme reduces financial hardship due to hospitalizations. Second, a key consideration is whether to extend existing insurance coverage to private sector drugs and outpatient care, but there are drawbacks to this policy. Such a programme will require additional financing and entail a substantial administrative burden to empanel and reimburse outpatient providers and chemist shops. Effectively making drugs free of charge could result in more low-value care, exacerbating the threat of antibiotic overuse. Instead, Odisha could consider altering incentives for providers and facilities. Incentivizing providers to ensure patients do not pay OOP for drugs (e.g. by using public pharmacies) could be a powerful way to counteract the kickbacks and other incentives that influence providers to prescribe drugs not in stock at public facilities (Kaplan *et al.*, 2012). Providers could be incentivized to persuade patients to use free, high-quality public sector drugs rather than the private sector. Public facilities are tasked with keeping drugs in stock, but there may not be consequences for low stocks. Incentives at the facility level for ensuring drugs are in stock and the drugs stocked are prescribed could be more effective policies than stricter enforcement of regulations.

### Limitations

We note limitations present in our data and analysis. First, all of our data are based on self-report, including where patients went for healthcare, the cause of the visit and how much was spent OOP; recall error could thus affect our results. Existing evidence indicates that respondents underestimate their healthcare utilization (Ansah and Powell-Jackson, 2013) but recall is better when individuals are sicker and, therefore, when costs are high (Das *et al*., 2011). Second, our survey did not ask hospitalized patients where they purchased their drugs and so we were unable to analyse the role of the private sector in drugs for hospitalized patients. Third, our data were collected over August–December 2019, and thus there could be some seasonal bias in our results. We validated our results extensively with the NSS to rule out seasonal effects, but there still remains some risk. Finally, our analyses are all associations, and thus none of our conclusions are causal in nature. Nonetheless, we believe our study provides strong evidence of the role played by the private sector in healthcare and drugs costs.

### Conclusion

To improve financial risk protection in Odisha, policymakers must consider the role of the private sector, and private chemist shops in particular, in contributing to financial hardship due to healthcare costs. Policy design should take into consideration the key function these providers currently serve as well as how existing shortcomings in the public sector provision of healthcare and insurance have resulted in high private sector OOP spending. Similar to other EAG states, Odisha faces substantial challenges in pursuing improved financial risk protection. With further health system analysis, policies suited to the specific challenges and attributes of Odisha can be designed to support the state in improving health system performance.

## Supplementary Material

czac035_Supp

## Data Availability

The data underlying this article will be shared on reasonable request to the corresponding author.

## Endnotes

1.
Table 1 shows drug spending accounts for 67% of CHE cases. Multiplying 65% by the assumed 86% of private sector drug use results in 56%. Private sector drug spending may make up more than 86% of drug OOP costs, however, and thus this is a conservative estimate of the potential impact of eliminating private sector drug costs on CHE.
