# Supplementary material for: Catastrophic health expenditure on private sector pharmaceuticals: a cross-sectional analysis from the state of Odisha, India
Source: Health Policy Plan. 2022 Apr 27;37(7):872–84. doi: 10.1093/heapol/czac035 (PMC9347020; doi:10.1093/heapol/czac035)
Supplement: czac035_Supp [file czac035_supp.zip › FRP Paper 1 Appendix Revised Clean.docx]

Appendix

Paper Title: Catastrophic health expenditure on private sector pharmaceuticals: A cross-sectional analysis from the state of Odisha, India

# Table of Contents

| **Section** | **Page** |
| --- | --- |
| Existing studies on Catastrophic Health Expenditure in India | 2 |
| Household Sampling | 19 |
| Weights Construction | 24 |
| Validation of the household survey with the National Sample Survey | 27 |
| Mapping of facility types | 31 |
| Mapping of reason for pursuing care | 32 |
|  |  |
| Decomposition analysis results | 34 |
|  |  |
| Essential medicine list stocks | 37 |
| Opening hours of different facility types | 41 |

# Existing studies on Catastrophic Health Expenditure in India

**Table A.1: Studies on Catastrophic Health Expenditure in India**

| **Number** | **Study** | **Outcome** | **Focus/breakdown** | **Analysis of drugs in CHE or OOP?** | **Analysis of private sector for drugs in CHE or OOP?** |
| --- | --- | --- | --- | --- | --- |
|  | Prinja et al. 2012 | OOP spending | Public versus private facilities  Poor versus non-poor | Yes | No |
|  | Dash & Mohanty 2019 | OOP spending | Poor versus non-poor | Yes | No |
|  | Ranjan et al. 2019 | OOP spending | Rheumatic diseases | Yes | No |
|  | Kundu et al. 2018 | OOP spending | NCDs | Yes | No |
|  | Tripathy et al. 2016 | OOP spending | NCDs | Yes | No |
|  | Thakur et al. 2011 | OOP spending | NCDs | Yes | No |
|  | Murphy et al. 2020 | OOP spending | NCDs | Yes | No |
|  | Engelgau et al. 2012 | OOP spending | NCDs | Yes | No |
|  | Kastor and Mohanty 2018 | OOP spending | NCDs, communicable diseases, and injuries | Yes | No |
|  | Selvaraj et al. 2018 | OOP spending | Multitude of disease areas | Yes | No |
|  | Sangar et al. 2019 | OOP spending | Multitude of disease areas | No | No |
|  | Tripathy & Prasad 2018 | OOP spending | Diabetes | No | No |
|  | Gwatidzo & Stewart 2017 | OOP spending | Diabetes | Yes | Yes |
|  | Singh et al. 2019 | OOP spending | Diabetes | No | No |
|  | Chauhan et al. 2019 | OOP & CHE | Head and neck cancer | Yes | No |
|  | Basavaiah et al. 2018 | OOP & CHE | Pancreatic cancer | Yes | No |
|  | Bradshaw et al. 2018 | OOP & CHE | Hemodialysis | Yes | No |
|  | Kaur et al. 2018 | OOP & CHE | Hemodialysis | Yes | No |
|  | Sarin et al. 2019 | OOP & CHE | Tuberculosis | Yes | No |
|  | Shewade et al. 2018 | OOP & CHE | Tuberculosis | Yes | No |
|  | Prasanna et al. 2018 | OOP & CHE | Tuberculosis | Yes | No |
|  | Yadav et al. 2019 | OOP & CHE | Tuberculosis | Yes | No |
|  | Kundu et al. 2015 | OOP & CHE | Tuberculosis | Yes | No |
|  | Muniyandi et al. 2020 | OOP & CHE | Tuberculosis | Yes | No |
|  | Mullerpattan et al. 2019 | OOP & CHE | Tuberculosis | Yes | Unable to verify |
|  | Poornima et al. 2020 | OOP & CHE | Tuberculosis | Yes | No |
|  | Chandra et al. 2020 | OOP & CHE | Tuberculosis | Yes | No |
|  | Mohanan et al. 2019 | OOP & CHE | Heart Disease | Yes | No |
|  | Alam & Mahal 2014 | OOP & CHE | Heart Disease | Yes | No |
|  | Huffman et al. 2011 | OOP & CHE | Heart Disease | Yes | No |
|  | Prinja et al. 2018 | OOP | Liver disorders | Yes | No |
|  | Rajpal et al. 2018 | CHE due to hospitalizations | Cancer | Yes | No |
|  | Pradhan et al. 2018 | CHE | Injuries | Yes | No |
|  | Tripathy et al. 2018 | CHE | Injuries | Yes | No |
|  | Prinja et al. 2016 | CHE | Injuries | Yes | No |
|  | Alam & Mahal 2016 | CHE | Injuries | Yes | No |
|  | Mohanty & Kastor 2017 | CHE | Pre/post National health mission | Yes | No |
|  | Dharmarajan et al. 2014 | CHE | Haemophilia | Yes | No |
|  | Ramachandran & Jha 2013 | CHE | Kidney transplants | Yes | No |
|  | Gopalan & Das 2009 | CHE | Other infectious diseases | Yes | No |
|  | Tripathy et al. 2017 | CHE | deliveries across private/public hospitalizations | Yes | No |
|  | Sahu & Bharati 2017 | OOP | Delivery, postpartum and neonatal health | Yes | No |
|  | Mishra & Mohanty 2019 | OOP | Delivery, postpartum and neonatal health | Yes | No |
|  | Sharma et al. 2018 | OOP | Delivery, postpartum and neonatal health | Yes | No |
|  | Agrawal et al. 2020 | OOP | Delivery, postpartum and neonatal health | Yes | No |
|  | Prinja et al. 2015 | OOP | Delivery, postpartum and neonatal health | Yes | No |
|  | Mohanty & Srivastava 2013 | OOP | Delivery, postpartum and neonatal health | Yes | No |
|  | Skordis-Worrall et al. 2011; | OOP | Delivery, postpartum and neonatal health | Yes | No |
|  | Bonu et al. 2009 | OOP | Delivery, postpartum and neonatal health | Yes | No |
|  | Goli et al. 2018 | OOP | Delivery, postpartum and neonatal health | Yes | No |
|  | Pandey et al. 2019 | CHE over time and across states | All | Yes | No |
|  | Bhojani et al. 2012 | CHE | Chronic conditions | Yes | No |
|  | Goeppel et al. 2016 | CHE | Chronic conditions | Yes | No |
|  | Haghparast-Bidgoli et al. 2015 | OOP | Sexual and reproductive health | Yes | No |
|  | Alvi et al. 2020 | CHE & OOP | HIV | Yes | No |
|  | Sharma et al. 2015 | CHE & OOP | HIV | Yes | No |
|  | Patel et al. 2007 | CHE & OOP | Other conditions | Yes | No |
|  | Savitha & Kiran 2015 | OOP | Health insurance status | Yes | No |
|  | Ranjan et al. 2017 | OOP | Hospitalization & insurance status | Yes | No |
|  | Nandi et al. 2017 | OOP | Hospitalization & insurance status | Yes | No |
|  | Wang et al. 2018 | CHE | Overall | Yes | No |
|  | Rahman et al. 2017 | CHE | Overall | Yes | No |
|  | Mohanty et al. 2017 | CHE | Overall | Yes | No |
|  | Pal 2012 | CHE | Overall | Yes | No |
|  | Taban et al. 2013 | CHE | Overall |  |  |
|  | Prinja et al. 2012 | CHE | Overall | Yes | No |
|  | Dwivedi & Pradhan 2020 | CHE | Overall | Yes | No |
|  | Shahrawat & Rao 2012 | CHE | Overall | Yes | No |
|  | Hadaye & Thampi 2018 | CHE | Inequities | Yes | No |
|  | Chowdhury et al. 2018 | CHE | Inequities | Yes | No |
|  | Sharma et al. 2017 | CHE | Inequities | Yes | No |
|  | Mohanty et al. 2018 | CHE | Inequities | Yes | No |
|  | Dhanaraj 2016 | CHE | Inequities | Yes | No |
|  | Karan et al. 2014 | CHE | Inequities | Yes | No |
|  | Kashyap et al. 2018 | CHE | Occupation | Yes | No |
|  | Lee et al. 2018 | CHE | Age | Yes | No |
|  | Brinda et al. 2015 | CHE | Age | Yes | No |
|  | Brinda et al. 2012 | CHE | Age | Yes | No |
|  | Singh et al. 2018 | CHE | Rural Areas | Yes | No |
|  | Loganathan et al. 2017 | CHE | Rural Areas | Yes | No |
|  | Sahu & Bharati 2017 | CHE | Urban Areas | Yes | No |
|  | Kusuma & Babu 2019 | CHE | Urban Areas | Yes | No |
|  | Seeberg et al. 2014 | CHE | Urban Areas | Yes | No |
|  | Gupt et al. 2016 | CHE | Among BPL beneficiaries and non-beneficiaries | Yes | Yes (in-house, out-house drugs) |
|  | Selvaraj et al. 2018 | CHE | Medicines only | Yes | No |
|  | Patil et al. 2009 | CHE | Childhood illness in an urban slum |  | No |
|  | Teixeira de Siqueira-Filha et al. 2021 | CHE | Urban Areas | No | No |
|  | Bhargava et al. 2020 | CHE | TB | No | No |
|  | Dwivedi et al. 2020 | CHE | Overall | No | No |
|  | Yadav et al. 2021 | OOP and CHE | TB | No | No |
|  | Rajasulochana & Kar 2021 | OOP and CHE | Stroke | Yes | No |
|  | Ecks 2021 | OOP and CHE | Insurance | No | No |
|  | Mendhe et al. 2021 | OOP and CHE | Insurance and urban areas | No | No |
|  | Raithatha et al. 2021 | OOP and CHE | West Syndrome | No | No |
|  | Verma et al. 2021 | OOP and CHE | NCD | Yes | No |
|  | Faizi et al. 2021 | OOP and CHE | Overall | No | No |
|  | CROCODILE study group 2021 | CHE | Colorectal Cancer | Yes | No |
|  | Yadav et al. 2021 | OOP and CHE | NCD | No | No |
|  | Dhankhar et al. 2021 | OOP and CHE | NCD | No | No |
|  | Joshi et al. 2020 | CHE | Insurance | No | No |
|  | Mohanty & Dwivedi 2021 | CHE | CHE methods | No | No |
|  | Goli et al. 2021 | OOP | Birth | No | No |
|  | Prasad et al. 2021 | CHE | TB | Yes | No |
|  | Yadav et al. 2021 | OOP and CHE | Overall | Yes | No |
|  | Yadav et al. 2021 | CHE | Injuries | Yes | No |
|  | Mahajan & Kaur 2021 | CHE | Rural Punjab | Yes | No |
|  | Dang et a. 2021 | OOP | Insurance | No | No |
|  | Balasundaram et al. 2020 | OOP | Hepatitis | Yes | No |
|  | Harish et al. 2020 | OOP | Insurance | Yes | No |
|  | Wu et al. 2020 | CHE | Cigarettes | No | No |
|  | Ranjan & Muraleedharan  2020 | CHE | Elderly | No | No |
|  | Singh et al 2020 | OOP and CHE | Cervical Cancer | Yes | No |
|  | Dalui et al. 2020 | OOP and CHE | Rural area | Yes | No |
|  | Kwesiga et al. 2020 | OOP | Insurance | Yes | No |
|  | Kumar et al. 2020 | CHE | Gender Differences | No | No |
|  | Pradhan & Behera 2021 | OOP and CHE | Child Care | No | No |
|  | Garg et al. 2020 | OOP and CHE | Insurance | No | No |
|  | Swetha et al. 2020 | OOP and CHE | Chronic Illness | Yes | No |
|  | Poornima et al. 2020 | OOP and CHE | TB | Yes | No |
|  | Dwivedi & Jalandhar 2020 | OOP | Overall | No | No |
|  | John et al. 2020 | OOP | Heart Failure | No | No |
|  | Rathi et al. 2020 | OOP | TB | Yes | No |
|  | Chandra et al. 2020 | OOP | TB | Yes | No |

Note: Analyzed is meant to capture whether the study represents private sector drug purchases, or proxies for that such as “outside drugs” or “pharmacy drugs” were represented in tables and figures or otherwise were separated out in quantitative and qualitative analysis. Studies that mention the role of the private sector in their background or interpretation of results were not marked as “Yes” because they did not do any analysis, even if they acknowledge the role of the private market as important.

**List of references for Table A.1**

Aggarwal, A. 2011. India Pharmaceuticals Sector. Credit Suisse. Available at: https://research-doc.credit-suisse.com/docView?language=ENG&source=emfromsendlink&format=PDF&document_id=919895261&serialid=N5pIkaBG%2BpA0BNF8wAjg3m5NlRbbZEZIYk8jiaMhMZI%3D (Accessed June 2020)

Aggarwal A. 2010. Impact evaluation of India's 'Yeshasvini' community-based health insurance programme. *Health Economics*. 19 Suppl:5‐35. doi:10.1002/hec.1605

Agrawal R, Negi R, Kaushal SK, Misra SK. 2020. Out of pocket expenditure and its associated factors in neonates admitted to neonatal intensive care unit of tertiary care government hospital of Agra District, Uttar Pradesh. *Indian Journal of Public Health*. 64(1):60‐65. doi:10.4103/ijph.IJPH_164_19

Ahlin T, Nichter M, Pillai G. 2016. Health insurance in India: what do we know and why is ethnographic research needed. *Anthropological Medicine*. 23(1):102‐124. doi:10.1080/13648470.2015.1135787

Alam K, Mahal A. 2014. The economic burden of angina on households in South Asia. *BMC Public Health*. 14:179. doi:10.1186/1471-2458-14-179

Alam K, Mahal A. 2016. The Economic Burden of Road Traffic Injuries on Households in South Asia. *PLoS One*. 11(10):e0164362. doi:10.1371/journal.pone.0164362

Alvi Y, Faizi N, Khalique N, Ahmad A. 2020. Assessment of out-of-pocket and catastrophic expenses incurred by patients with Human Immunodeficiency Virus (HIV) in availing free antiretroviral therapy services in India. *Public Health*. 2020;183:16‐22. doi:10.1016/j.puhe.2020.03.031

Balasubramanian D, Prinja S, Aggarwal AK. 2015. Effect of User Charges on Secondary Level Surgical Care Utilization and Out-of-Pocket Expenditures in Haryana State, India. *PLoS One*. 2015;10(5):e0125202. doi:10.1371/journal.pone.0125202

Balasundaram P, Tiwari VK, Sherin Raj TP. 2020. Cost of treatment and consequences for chronic hepatitis B and C virus infection at a tertiary care hospital in Delhi. *Indian Journal Public Health.* doi: 10.4103/ijph.IJPH_356_19.

Basavaiah G, Rent PD, Rent EG, Sullivan R, Towne M, Bak M, Sirohi B, Goel M, Shrikhande SV. 2018. Financial Impact of Complex Cancer Surgery in India: A Study of Pancreatic Cancer. *Journal of Global Oncology*. 2018 Sep;4:1-9. doi: 10.1200/JGO.17.00151.

Bergkvist S, Wagstaff A, Katyal A, Singh PB, Samarth A, Rao M. 2014. What a Difference a State Makes: Health Reform in Andhra Pradesh. The World Bank: Development Research Group. Human Development and Public Services Team. Policy Research Paper 6883.

Bhargava A, Bhargava M, Meher A. 2020. Universal health coverage and tuberculosis care in India in the times of Covid-19: Aligning Ayushman Bharat (National Health Assurance Scheme) to improve case detection, reduce deaths and catastrophic health expenditure. *National Medical Journal of India.* 33(5):298-301. doi: 10.4103/0970-258X.303111.

Bhojani U, Thriveni B, Devadasan R, et al. 2012. Out-of-pocket healthcare payments on chronic conditions impoverish urban poor in Bangalore, India. *BMC Public Health*. 12:990. doi:10.1186/1471-2458-12-990

Bonu S, Bhushan I, Rani M, Anderson I. 2009. Incidence and correlates of 'catastrophic' maternal health care expenditure in India. *Health Policy and Planning*. 24(6):445‐456. doi:10.1093/heapol/czp032

Boswell Dean E. 2019. Who Benefits from Pharmaceutical Price Controls? Evidence from India . CGD Working Paper 509. Washington, DC: Center for Global Development. Available at: https://www.cgdev.org/publication/who-benefits-pharmaceutical-pricecontrols-

evidence-india (Accessed May 2020)

Bradshaw C, Gracious N, Narayanan R, Narayanan S, Safeer M, Nair GM, Murlidharan P, Sundaresan A, Retnaraj Santhi S, Prabhakaran D, Kurella Tamura M, Jha V, Chertow GM, Jeemon P, Anand S. 2018. Paying for Hemodialysis in Kerala, India: A Description of Household Financial Hardship in the Context of Medical Subsidy. *Kidney International Reports.* 4(3):390-39

Brinda EM, Kowal P, Attermann J, Enemark U. 2015. Health service use, out-of-pocket payments and catastrophic health expenditure among older people in India: the WHO Study on global AGEing and adult health (SAGE). *Journal of Epidemiology and Community Health*. 69(5):489‐494. doi:10.1136/jech-2014-204960

Brinda EM, Rajkumar AP, Enemark U, Prince M, Jacob KS. 2012. Nature and determinants of out-of-pocket health expenditure among older people in a rural Indian community. *International Psychogeriatrics*. 2012;24(10):1664‐1673. doi:10.1017/S104161021200083X

Chandra A, Kumar R, Kant S, Parthasarathy R, Krishnan A. 2020. Direct and indirect patient costs of tuberculosis care in India. *Tropical Medicine and International Health.* 25(7):803-812. doi: 10.1111/tmi.13402.

Chandra A, Kumar R, Kant S, Parthasarathy R, Krishnan A. 2020. Direct and indirect patient costs of tuberculosis care in India. *Tropical Medicine and International Health*. 10.1111/tmi.13402. doi:10.1111/tmi.13402

Chauhan AS, Prinja S, Ghoshal S, Verma R. 2019. Economic Burden of Head and Neck Cancer Treatment in North India. *Asian Pacific Journal of Cancer Prevention.* 20(2):403-409.

Chowdhury S, Gupta I, Trivedi M, Prinja S. 2018. Inequity & burden of out-of-pocket health spending: District level evidences from India. Indian J Med Res. 148(2):180-189. doi: 10.4103/ijmr.IJMR_90_17.

Dang A, Dang D, Vallish BN. 2021. Importance of Evidence-Based Health Insurance Reimbursement and Health Technology Assessment for Achieving Universal Health Coverage and Improved Access to Health in India. *Value in Health Regional Issues.* 24:24-30. doi: 10.1016/j.vhri.2020.04.007.

[Dash](https://www.ncbi.nlm.nih.gov/pubmed/?term=Dash%20A%5BAuthor%5D&cauthor=true&cauthor_uid=31362727) A, [Mohanty](https://www.ncbi.nlm.nih.gov/pubmed/?term=Mohanty%20SK%5BAuthor%5D&cauthor=true&cauthor_uid=31362727) SK. 2019. Do poor people in the poorer states pay more for healthcare in India? [*BMC Public Health*](https://www.ncbi.nlm.nih.gov/pmc/articles/PMC6668144/)*.* 19:1020.

Dalui A, Banerjee S, Roy R. 2020. Determinants of out-of-pocket and catastrophic health expenditure in rural population: A community-based study in a block of Purba Barddhaman, West Bengal. *Indian Journal of Public Health.* 64(3):223-228. doi: 10.4103/ijph.IJPH_848_20.

Dhanaraj S. 2016. Economic vulnerability to health shocks and coping strategies: evidence from Andhra Pradesh, India. *Health Policy and Planning*. 31(6):749‐758. doi:10.1093/heapol/czv127

Dharmarajan S, Phadnis S, Gund P, Kar A. 2014. Out-of-pocket and catastrophic expenditure on treatment of haemophilia by Indian families. *Haemophilia*. 20(3):382‐387. doi:10.1111/hae.12324

Directorate General of Health Services. 2012. Indian Public Health Standards (IPHS) guidelines for primary health centres revised 2012. Available at: http://nhm.gov.in/images/pdf/guidelines/iphs/iphs-revised-guidlines-2012/primay-health-centres.pdf  (Accessed June 2020)

Devadasan N, Criel B, Van Damme W, Ranson K, Van der Stuyft P. 2007. Indian community health insurance schemes provide partial protection against catastrophic health expenditure. *BMC Health Services Research*. 7:43. doi:10.1186/1472-6963-7-43

Devadasan N, Seshadri T, Trivedi M, et al. 2013. Promoting universal financial protection: evidence from the Rashtriya Swasthya Bima Yojana (RSBY) in Gujarat, India. *Health Research Policy and Systems*. 11:1–29. 10.1186/1478-4505-11-29

Dhanaraj S. 2014. Health shocks and coping strategies: State health insurance scheme of Andhra Pradesh, India. World Institute for Development Economics Research (WIDER) Working Paper 2014/003.

Dwivedi R, Pradhan J. 2020. Does affordability matter? Examining the trends and patterns in health care expenditure in India. *Health Services Management Research.* 33(4):207-218. doi: 10.1177/0951484820923921.

Dwivedi R, Pradhan J. 2020. Does affordability matter? Examining the trends and patterns in health care expenditure in India.  *Health Services Management Research*. 951484820923921. doi:10.1177/0951484820923921

Dwivedi R, Pradhan J, Athe R. 2021. Measuring catastrophe in paying for healthcare: A comparative methodological approach by using National Sample Survey, India. *International Journal of Health Planning and Management*. doi: 10.1002/hpm.3272.

Ecks S. 2021. "Demand Side" Health Insurance in India: The Price of Obfuscation*. Medical Anthropology.* 40(5):404-416. doi: 10.1080/01459740.2021.1929208

Engelgau MM, Karan A, Mahal A. 2012. The Economic impact of Non-communicable Diseases on households in India. *Global Health*. 8:9. doi:10.1186/1744-8603-8-9

Faizi N, Alvi Y. 2021. Comment on: “Disease-Specific Out-of-Pocket Payments, Catastrophic Health Expenditure and Impoverishment Effects in India: An Analysis of National Health Survey Data”.*Applied Health Economics and Health Policy*.

Fan VY, Karan A, Mahal A. 2012. State health insurance and out-of-pocket health expenditures in Andhra Pradesh, India. *International Journal of Health Care Finance and Economics*. 12(3):189‐215. doi:10.1007/s10754-012-9110-5

Garg S, Bebarta KK, Tripathi N. 2020. Performance of India's national publicly funded health insurance scheme, Pradhan Mantri Jan Arogaya Yojana (PMJAY), in improving access and financial protection for hospital care: findings from household surveys in Chhattisgarh state. *BMC Public Health.* 20(1):949. doi: 10.1186/s12889-020-09107-4.

Garg S, Chowdhury S, Sundararaman T. 2019. Utilisation and financial protection for hospital care under publicly funded health insurance in three states in Southern India. *BMC Health Services Research*. 19(1):1004. doi:10.1186/s12913-019-4849-8

Goeppel C, Frenz P, Grabenhenrich L, Keil T, Tinnemann P. 2016. Assessment of universal health coverage for adults aged 50 years or older with chronic illness in six middle-income countries. *Bulletin of the World Health Organization*. 94(4):276‐85C. doi:10.2471/BLT.15.163832

Goli S, Moradhvaj, Pradhan J, Reja T. 2021. The unending burden of high out-of-pocket expenditure on institutional deliveries in India. *Public Health.* 193:43-47. doi: 10.1016/j.puhe.2021.01.029.

Goli S, Rammohan A, Moradhvaj. 2018. Out-of-pocket expenditure on maternity care for hospital births in Uttar Pradesh, India. *Health Economics Review.* 8(1):5. doi: 10.1186/s13561-018-0189-3.

Gopalan SS, Das A. 2009. Household economic impact of an emerging disease in terms of catastrophic out-of-pocket health care expenditure and loss of productivity: investigation of an outbreak of chikungunya in Orissa, India. *Journal of Vector Borne Diseases*. 46(1):57‐64.

Grassi S, Albert Ma CT. 2012. Public Sector Rationing and Private Sector Selection. *Journal of Public Economic Theory*. 14(1): 1-34.

Gupt A, Kaur P, Kamraj P, Murthy BN. 2016. Out of Pocket Expenditure for Hospitalization among Below Poverty Line Households in District Solan, Himachal Pradesh, India, 2013. *PLoS One*. 11(2):e0149824. doi:10.1371/journal.pone.0149824

Gwatidzo SD, Stewart Williams J. 2017. Diabetes mellitus medication use and catastrophic healthcare expenditure among adults aged 50+ years in China and India: results from the WHO study on global AGEing and adult health (SAGE). *BMC Geriatrics.* 17(1):14. doi: 10.1186/s12877-016-0408-x.

Hadaye RS, Thampi JG. 2018. Catastrophic Health-care Expenditure and Willingness to Pay for Health Insurance in a Metropolitan City: A Cross-Sectional Study. *Indian Journal of Community Medicine.* 43(4):307-311. doi: 10.4103/ijcm.IJCM_252_18.

Haghparast-Bidgoli H, Pulkki-Brännström AM, Lafort Y, et al. 2015. Inequity in costs of seeking sexual and reproductive health services in India and Kenya. *International Journal of Equity and Health*. 14:84. doi:10.1186/s12939-015-0216-5

Harish R, Suresh RS, Rameesa S, Laiveishiwo PM, Loktongbam PS, Prajitha KC, Valamparampil MJ. 2020. Health insurance coverage and its impact on out-of-pocket expenditures at a public sector hospital in Kerala, India. *Journal of Family Medicine and Primaru Care.* _ 665:20. doi: 10.4103/jfmpc.jfmpc

Huffman MD, Rao KD, Pichon-Riviere A, et al. 2011. A cross-sectional study of the microeconomic impact of cardiovascular disease hospitalization in four low- and middle-income countries. *PLoS One*. 6(6):e20821. doi:10.1371/journal.pone.0020821

John KJ, Turaka VP, Muruga Bharathy K, Vignesh Kumar C, Jayaseelan L, Visalakshi J, Nadaraj A, Mathew A, Mariam F, Nellimala NJ, Joy A, Punitha JV, Koshy M, Chandy G, Gunasekaran K, Sudarsanam TD. 2020. Predictors of mortality, strategies to reduce readmission, and economic impact of acute decompensated heart failure: Results of the Vellore Heart Failure Registry. *Indian Heart Journal.* 72(1):20-26. doi: 10.1016/j.ihj.2020.03.005.

Joshi R, Pakhare A, Yelwatkar S, Bhan A, Kalantri SP, Jajoo UN. 2020. Impact of community-based health insurance and economic status on utilization of healthcare services: A household-level cross-sectional survey from rural central India. *National Medical Journal of India.* 33(2):74-82. doi: 10.4103/0970-258X.310921.

Katyal A, Singh PV, Bergkvist S, Samarth A, Rao M. 2015. Private sector participation in delivery tertiary health care: a dichotomy of access and affordability across two Indian states. *Health Policy and Planning.* 30:i23-i31.

# Karan A, Yip W, Mahal A. 2017. Extending health insurance to the poor in India: An impact evaluation of Rashtriya Swasthya Bima Yojana on out of pocket spending for healthcare. *Social Science & Medicine.* 181:83-92.

Karan A, Selvaraj S, Mahal A. 2014. Moving to universal coverage? Trends in the burden of out-of-pocket payments for health care across social groups in India, 1999-2000 to 2011-12. *PLoS One*. 9(8):e105162. doi:10.1371/journal.pone.0105162

Kashyap GC, Singh SK, Sharma SK. 2018. Catastrophic Health Expenditure and Impoverishment Effects of Out-of-pocket Expenses: A Comparative Study of Tannery and Non-tannery Workers of Kanpur, India. *Indian Journal of Occupational and Environmental Medicine.* 22(1):22-28. doi: 10.4103/ijoem.IJOEM_168_17.

Kastor A, Mohanty SK. 2018. Disease-specific out-of-pocket and catastrophic health expenditure on hospitalization in India: Do Indian households face distress health financing? *PLoS One.* 13(5):e0196106.

Kaur G, Prinja S, Ramachandran R, Malhotra P, Gupta KL, Jha V . 2018. Cost of hemodialysis in a public sector tertiary hospital of India. *Clinical Kidney Journal.* 11(5):726-733. doi: 10.1093/ckj/sfx152.

Kumar K, Singh A, James KS, McDougal L, Raj A. 2020. Gender bias in hospitalization financing from borrowings, selling of assets, contribution from relatives or friends in India. *Social Science and Medicine.* 260:113222. doi: 10.1016/j.socscimed.2020.113222.

Kundu MK, Hazra S, Pal D, Bhattacharya M. 2018. A review on Noncommunicable Diseases (NCDs) burden, its socio-economic impact and the strategies for prevention and control of NCDs in India. *Indian Journal of Public Health*. 62(4):302‐304. doi:10.4103/ijph.IJPH_324_16

Kundu D, Katre V, Singh K, et al. 2015. Innovative social protection mechanism for alleviating catastrophic expenses on multidrug-resistant tuberculosis patients in Chhattisgarh, India. *WHO South East Asia Journal of Public Health*. 4(1):69‐77. doi:10.4103/2224-3151.206624

Kusuma YS, Babu BV. 2019. The costs of seeking healthcare: Illness, treatment seeking and out of pocket expenditures among the urban poor in Delhi, India. *Health and Social Care in the Community*. doi: 10.1111/hsc.12792.

Kwesiga B, Aliti T, Nabukhonzo P, Najuko S, Byawaka P, Hsu J, Ataguba JE, Kabaniha G. What has been the progress in addressing financial risk in Uganda? Analysis of catastrophe and impoverishment due to health payments. *BMC Health Services Research.* 20(1):741. doi: 10.1186/s12913-020-05500-2. Erratum in: *BMC Health Services Research.* 20(1):843.

Laine LT, Ma CA. 2017. Quality and competition between public and private firms. *Journal of Economic Behavior & Organization*. 140(C): 336-353.

Lee TJ, Saran I, Rao KD. 2018. Ageing in India: Financial hardship from health expenditures. *International Journal of Health Planning and Management.* 33(2):414-425. doi: 10.1002/hpm.2478.

Loganathan K, Deshmukh PR, Raut AV. 2017. Socio-demographic determinants of out-of-pocket health expenditure in a rural area of Wardha district of Maharashtra, India. *Indian Journal of Medical Research.* 146(5):654-661. doi: 10.4103/ijmr.IJMR_256_15.

Lim SS, Dandona L, Hoisington JA, James SL, Hogan MC, Gakidou E. 2010. India’s Janani Suraksha Yojana, a conditional cash transfer programme to increase births in health facilities: an impact evaluation. *The Lancet.* 375(9730):2009–2023.

Mahajan N, Kaur B. 2021. Analysing the expenditure on childbearing: a community-based cross-sectional study in rural areas of Punjab (India). *BMC Health Services Research*.21:76. doi: 10.1186/s12913-021-06075-2.

Mendhe HG, David R, Singh D, Makade KG. 2021. Universal Health Insurance coverage and utilization among women in urban slum of Rajnandgaon, Chhattisgarh. *Journal of Family Medicine and Primary Care.* 10(3):1313-1319. doi: 10.4103/jfmpc.jfmpc_2226_20.

[Mishra S](https://www.ncbi.nlm.nih.gov/pubmed/?term=Mishra%20S%5BAuthor%5D&cauthor=true&cauthor_uid=31238928), [Mohanty SK](https://www.ncbi.nlm.nih.gov/pubmed/?term=Mohanty%20SK%5BAuthor%5D&cauthor=true&cauthor_uid=31238928). 2019. Out-of-pocket expenditure and distress financing on institutional delivery in India. [*International Journal of Equity in Health.*](https://www.ncbi.nlm.nih.gov/pubmed/31238928) 18(1):99.

Mohanan PP, Huffman MD, Baldridge AS, Devarajan R, Kondal D, Zhao L, Ali M, Joseph J, Eapen K, Krishnan MN, Menon J, Thomas M, Lloyd-Jones DM, Harikrishnan S, Prabhakaran D; ACS QUIK Investigators. 2019. Microeconomic Costs, Insurance, and Catastrophic Health Spending Among Patients With Acute Myocardial Infarction in India: Substudy of a Randomized Clinical Trial. *JAMA Network Open.* 2(5):e193831. doi: 10.1001/jamanetworkopen.2019.3831.

Mohanty SK, Dwivedi LK. 2021. Addressing data and methodological limitations in estimating catastrophic health spending and impoverishment in India, 2004-18. *International Journal for Equity in Health.* 20(1):85. doi: 10.1186/s12939-021-01421-6.

Mohanty SK, Kastor A. 2017. Out-of-pocket expenditure and catastrophic health spending on maternal care in public and private health centres in India: a comparative study of pre and post national health mission period. *Health Economics Review.* 7(1):31.

Mohanty SK, Agrawal NK, Mahapatra B, Choudhury D, Tuladhar S, Holmgren EV. 2017. Multidimensional poverty and catastrophic health spending in the mountainous regions of Myanmar, Nepal and India. Int J Equity Health.16(1):21. doi: 10.1186/s12939-016-0514-6.

Mohanty SK, Kim R, Khan PK, Subramanian SV. 2018. Geographic Variation in Household and Catastrophic Health Spending in India: Assessing the Relative Importance of Villages, Districts, and States, 2011-2012. Milbank Q. 96(1):167-206. doi: 10.1111/1468-0009.12315.

Mohanty SK, Srivastava A. 2013. Out-of-pocket expenditure on institutional delivery in India. *Health Policy and Planning*. 28(3):247‐262. doi:10.1093/heapol/czs057

Mukherjee S, Singh A. 2018. Has the *Janani Suraksha Yojana* (a conditional maternity benefit transfer scheme) succeeded in reducing the economic burden of maternity in rural India? Evidence from the Varanasi district of Uttar Pradesh. *Journal of Public Health Research.* 7(1):957. doi: 10.4081/jphr.2018.957. eCollection 2018 Feb 5.

Mullerpattan JB, Udwadia ZZ, Banka RA, Ganatra SR, Udwadia ZF. 2019. Catastrophic costs of treating drug resistant TB patients in a tertiary care hospital in India. *Indian Journal of Tuberculosis*. 66(1):87‐91. doi:10.1016/j.ijtb.2018.04.011

Muniyandi M, Thomas BE, Karikalan N, et al. 2020. Association of Tuberculosis With Household Catastrophic Expenditure in South India. *JAMA Network Open*. 3(2):e1920973. doi:10.1001/jamanetworkopen.2019.20973

Murphy A, Palafox B, Walli-Attaei M, et al. 2020. The household economic burden of non-communicable diseases in 18 countries. *BMJ Global Health*. 5(2):e002040. doi:10.1136/bmjgh-2019-002040

Nandi S, Schneider H, Dixit P. 2017. Hospital utilization and out of pocket expenditure in public and private sectors under the universal government health insurance scheme in Chhattisgarh State, India: Lessons for universal health coverage. *PLoS One.* 12(11):e0187904. doi: 10.1371/journal.pone.0187904.

Pal R. 2021. Measuring incidence of catastrophic out-of-pocket health expenditure: with application to India. *International Journal of Health Care Finance and Economics*. 12(1):63‐85. doi:10.1007/s10754-012-9103-4

Pandey A, Kumar GA, Dandona R, Dandona L. 2018. Variations in catastrophic health expenditure across the states of India: 2004 to 2014. *PLoS One.* 13(10):e0205510. doi: 10.1371/journal.pone.0205510.

Patel V, Chisholm D, Kirkwood BR, Mabey D. 2007. Prioritizing health problems in women in developing countries: comparing the financial burden of reproductive tract infections, anaemia and depressive disorders in a community survey in India. *Tropical Medicine and International Health*. 12(1):130‐139. doi:10.1111/j.1365-3156.2006.01756.x

Patil SS, Berad AS, Angadi MM. 2009. A study to assess catastrophic household expenditure on childhood illness in an urban slum in bijapur. *Indian Journal of Community Medicine*. 34(4):335‐337. doi:10.4103/0970-0218.58394.

Poornima MP, Shruthi MN, Chingale AL, Veena V, Nagaraja SB, Madhukeshwar AK. 2020. Cost of Tuberculosis Care in Programmatic Settings from Karnataka, India: Is It Catastrophic for the Patients?. *Tuberculosis Research and Treatment*. 2020:3845694. doi:10.1155/2020/3845694

Pradhan J, Behera S. 2020. Does choice of health care facility matter? Assessing out-of-pocket expenditure and catastrophic spending on emergency obstetric care in India. *Journal of Biosocial Science.* 2021 Jul;53(4):481-496. doi: 10.1017/S0021932020000310.

Pradhan J, Dwivedi R, Pati S, Rout SK. 2017. Does spending matters? Re-looking into various covariates associated with Out of Pocket Expenditure (OOPE) and catastrophic spending on accidental injury from NSSO 71st round data. *Health Economics Review.* 7(1):48.

Prasad BM, Tripathy JP, Muraleedharan VR, Tonsing J. 2021. Rising Catastrophic Expenditure on Households Due to Tuberculosis: Is India Moving Away From the END-TB Goal? *Front Public Health.* 9:614466. doi: 10.3389/fpubh.2021.614466.

Prasanna T, Jeyashree K, Chinnakali P, Bahurupi Y, Vasudevan K, Das M. 2018. Catastrophic costs of tuberculosis care: a mixed methods study from Puducherry, India. *Global Health Action.* 11(1):1477493. doi: 10.1080/16549716.2018.1477493.

Prinja S, Kaur M, Kumar R. 2012. Universal health insurance in India: ensuring equity, efficiency, and quality. *Indian Journal of Community Medicine*. 37(3):142‐149. doi:10.4103/0970-0218.99907

Prinja S, Bahuguna P, Gupta R, Sharma A, Rana SK, Kumar R. 2015. Coverage and Financial Risk Protection for Institutional Delivery: How Universal Is Provision of Maternal Health Care in India?. *PLoS One*. 10(9):e0137315. Published 2015 Sep 8. doi:10.1371/journal.pone.0137315

Prinja S, Bahuguna P, Gupta I, Chowdhury S, Trivedi M. 2019. Role of insurance in determining utilization of healthcare and financial risk protection in India. PLoS One. 14(2):e0211793. doi: 10.1371/journal.pone.0211793.

Prinja S, Jagnoor J, Chauhan AS, Aggarwal S, Nguyen H, Ivers R. 2016. Economic Burden of Hospitalization Due to Injuries in North India: A Cohort Study. *International Journal of Environmental Research and Public Health*. 13(7):673. doi:10.3390/ijerph13070673

Prinja S, Kanavos P, Kumar R. 2012. Health care inequities in north India: role of public sector in universalizing health care. *Indian Journal of Medical Research*. 136(3):421‐431.

Prinja S, Bahuguna P, Duseja A, Kaur M, Chawla YK. 2018. Cost of Intensive Care Treatment for Liver Disorders at Tertiary Care Level in India. *PharmacoEconomics Open*.  2(2):179-190. doi: 10.1007/s41669-017-0041-4.

Prinja S, Aggarwal AK, Kumar R, Kanavos P. 2012. User charges in health care: evidence of effect on service utilization & equity from north India. *Indian Journal of Medical Research*. 136(5):868‐876.

Prinja S, Chauhan AS, Karan A, et al. 2017. Impact of publicly financed health insurance schemes on healthcare utilization and financial risk protection in india: a systematic review. *PLoS One.*  12:e0170996.

Quintussi M, Van de Poel E, Panda P, Rutten F. 2015. Economic consequences of ill-health for households in northern rural India. *BMC Health Services Research*. 15:179. doi:10.1186/s12913-015-0833-0

Raban MZ, Dandona R, Dandona L. 2013. Variations in catastrophic health expenditure estimates from household surveys in India. *Bulletin of the World Health Organization*. 91(10):726‐735. doi:10.2471/BLT.12.113100

Rahman MM, Karan A, Rahman MS, Parsons A, Abe SK, Bilano V, Awan R, Gilmour S, Shibuya K. 2017. Progress Toward Universal Health Coverage: A Comparative Analysis in 5 South Asian Countries. *JAMA Internal Medicine.* 177(9):1297-1305. doi: 10.1001/jamainternmed.2017.3133.

Raithatha D, Sahu JK, Bhanudeep S, Saini L, Prinja S, Bharti B, Madaan P, Rana SK, Negi S. 2021. Financial Burden in Families of Children with West Syndrome. *Indian Journal of Pediatrics.* doi: 10.1007/s12098-021-03761-1.

Rajasulochana SR, Kar SS. 2021. Economic burden associated with stroke in India: insights from national sample survey 2017-18. *Expert Review of Pharmacoeconomics and Outcomes Research.* doi: 10.1080/14737167.2021.1941883.

Rajpal S, Kumar A, Joe W. 2018. Economic burden of cancer in India: Evidence from cross-sectional nationally representative household survey, 2014. *PLoS One.* 13(2):e0193320.

Ramachandran R, Jha V. 2013. Kidney transplantation is associated with catastrophic out of pocket expenditure in India. *PLoS One*. 8(7):e67812. doi:10.1371/journal.pone.0067812

Ranjan A, Muraleedharan VR. 2020. Equity and elderly health in India: reflections from 75th round National Sample Survey, 2017-18, amidst the COVID-19 pandemic. *Global Health.* 16(1):93. doi: 10.1186/s12992-020-00619-7.

Ranjan A, Thiagarajan S, Garg S, Danda D. 2019. Progress towards universal health coverage in the context of rheumatic diseases in India.  *International Journal of Rheumatic Diseases.* 22(5):880-889.

Ranjan A, Dixit P, Mukhopadhyay I, Thiagarajan S. 2018. Effectiveness of government strategies for financial protection against costs of hospitalization Care in India. *BMC Public Health.* 18(1):501. doi: 10.1186/s12889-018-5431-8.

Ranson MK. 2002. Reduction of catastrophic health care expenditures by a community-based health insurance scheme in Gujarat, India: current experiences and challenges. *Bulletin of the World Health Organization*. 80(8):613‐621.

Rathi P, Shringarpure K, Unnikrishnan B, Chadha VK, Acharya V, Nair A, Sagili KD, Shastri S. 2020. Pretreatment Out-of-Pocket Expenses for Presumptive Multidrug-Resistant Tuberculosis Patients, India, 2016-2017. *Emerging Infectious Disease.* 26(5):989-992. doi: 10.3201/eid2605.181992.

Rao M, Katyal A, Singh PV, Smarth A, Bergkvist S, Kancharla M, Wagstaff A, Netuveli G, Renton A. 2014. Changes in addressing inequalities in access to hospital care in Andhra Pradesh and Maharashtra states of India: a difference-in-differences study using repeated cross-sectional surveys. *BMJ Open.* 4:e004471.

Roy, V., Gupta, U., & Agarwal, A. K. 2012. Cost of medicines & their affordability in private pharmacies in Delhi (India). *Indian Journal of Medical Research*. 136(5): 827-835.

Sahu KS, Bharati B. Out-of-Pocket health expenditure and sources of financing for delivery, postpartum, and neonatal health in urban slums of Bhubaneswar, Odisha, India. *Indian Journal of Public Health.* 2017; 61: 67-73.

Sangar S, Dutt V, Thakur R. 2019. Comparative Assessment of Economic Burden of Disease in Relation to Out of Pocket Expenditure. *Front Public Health.* 7:9. doi: 10.3389/fpubh.2019.00009.

Sarin R, Vohra V, Singla N, Thomas B, Krishnan R, Muniyandi M. 2019. Identifying costs contributing to catastrophic expenditure among TB patients registered under RNTCP in Delhi metro city in India. *Indian Journal of Tuberculosis.* 66(1):150-157. doi: 10.1016/j.ijtb.2018.10.009.

Savitha S, Kiran KB. 2015. Effectiveness of micro health insurance on financial protection: Evidence from India. *International Journal of Health Economics and Management*. 15(1):53‐71. doi:10.1007/s10754-014-9158-5

Seeberg J, Pannarunothai S, Padmawati RS, Trisnantoro L, Barua N, Pandav CS. 2014. Treatment seeking and health financing in selected poor urban neighbourhoods in India, Indonesia and Thailand. *Social Science and Medicine*. 102:49‐57. doi:10.1016/j.socscimed.2013.11.039

Selvaraj S, Farooqui HH, Karan A. 2018. Quantifying the financial burden of households' out-of-pocket payments on medicines in India: a repeated cross-sectional analysis of National Sample Survey data, 1994-2014. *BMJ Open*. 8(5):e018020.

Selvaraj S, Karan AK. 2012. Why Publicly-Financed Health Insurance Schemes Are Ineffective in Providing Financial Risk Protection. *Economic & Political Weekly.* XLVII: 11.

Sharma V, Krishnaswamy D, Mulay S. 2015. Consumption patterns and levels among households with HIV positive members and economic impoverishment due to medical spending in Pune city, India. *AIDS Care*. 27(7):916‐920. doi:10.1080/09540121.2015.1015482

Sharma S, Verma PB, Viramgami AP, Vala MC, Lodhiya KK. 2018. Analysis of Out-of-Pocket Expenditure in Utilization of Maternity Care Services in Urban Slums of Rajkot City, Gujarat. *Indian Journal of Community Medicine*. 43(3):215-219. doi: 10.4103/ijcm.IJCM_47_18.

Sharma D, Prinja S, Aggarwal AK, Bahuguna P, Sharma A, Rana SK. 2017. Out-of-pocket expenditure for hospitalization in Haryana State of India: Extent, determinants & financial risk protection. *Indian Journal of Medical Research.*  146(6):759-767. doi: 10.4103/ijmr.IJMR_2003_15.

Shahrawat R, Rao KD. 2012. Insured yet vulnerable: out-of-pocket payments and India's poor. *Health Policy and Planning*. 27(3):213‐221. doi:10.1093/heapol/czr029

Shewade HD, Gupta V, Satyanarayana S, Kharate A, Sahai KN, Murali L, Kamble S, Deshpande M, Kumar N, Kumar S, Pandey P, Bajpai UN, Tripathy JP, Kathirvel S, Pandurangan S, Mohanty S, Ghule VH, Sagili KD, Prasad BM, Nath S, Singh P, Singh K, Singh R, Jayaraman G, Rajeswaran P, Srivastava BK, Biswas M, Mallick G, Bera OP, Jaisingh AJJ, Naqvi AJ, Verma P, Ansari MS, Mishra PC, Sumesh G, Barik S, Mathew V, Lohar MRS, Gaurkhede CS, Parate G, Bale SY, Koli I, Bharadwaj AK, Venkatraman G, Sathiyanarayanan K, Lal J, Sharma AK, Rao R, Kumar AMV, Chadha SS; as members of the Axshya SAMVAD study group. 2018. Active case finding among marginalised and vulnerable populations reduces catastrophic costs due to tuberculosis diagnosis. *Global Health Action.* 11(1):1494897. doi: 10.1080/16549716.2018.1494897.

Singh MP, Chauhan AS, Rai B, Ghoshal S, Prinja S. 2020. Cost of Treatment for Cervical Cancer in India. *Asian Pacific Journal of Cancer Prevention.* 21(9):2639-2646. doi: 10.31557/APJCP.2020.21.9.2639. PMID: 32986363.

Singh T, Bhatnagar N, Singh G, Kaur M, Kaur S, Thaware P, Kumar R. 2018. Health-care utilization and expenditure patterns in the rural areas of Punjab, India. *Journal of Family Medicine and Primary Care.* 7(1):39-44. doi: 10.4103/jfmpc.jfmpc_291_17.

Singh PV, Tatambhotla A, Kalvakuntla R, et al. 2013. Understanding public drug procurement in India: a comparative qualitative study of five Indian states. *BMJ Open.* 3:e001987

Singh K, Narayan KMV, Eggleston K. 2019. Economic Impact of Diabetes in South Asia: the Magnitude of the Problem. *Current Diabetes Reports*. 19(6):34. doi:10.1007/s11892-019-1146-1

Skordis-Worrall J, Pace N, Bapat U, et al. 2011. Maternal and neonatal health expenditure in Mumbai slums (India): a cross sectional study. *BMC Public Health*. 11:150. doi:10.1186/1471-2458-11-150

Sood N, Bendavid E, Mukherji A, Wagner Z, Nagpal S, Mullen P. 2014. Government health insurance for people below poverty line in India quasi-experimental evaluation of insurance and health outcomes. *BMJ.* 349:g5114.

Swetha NB, Shobha S, Sriram S. 2020. Prevalence of catastrophic health expenditure and its associated factors, due to out-of-pocket health care expenses among households with and without chronic illness in Bangalore, India: a longitudinal study. *Journal of Preventive Medicine and Hygiene.* 61(1):E92-E97. doi: 10.15167/2421-4248/jpmh2020.61.1.1191.

Teixeira de Siqueira-Filha N, Li J, Kibuchi E*, et al* 2020. Economics of healthcare access in low-income and middle-income countries: a protocol for a scoping review of the economic impacts of seeking healthcare on slum-dwellers compared with other city residents. *BMJ Open*. 11**:**e045441. doi: 10.1136/bmjopen-2020-045441

Thakur J, Prinja S, Garg CC, Mendis S, Menabde N. 2011. Social and Economic Implications of Noncommunicable diseases in India. *Indian Journal of Community Medicine*. 36(Suppl 1):S13‐S22. doi:10.4103/0970-0218.94704

Tripathi N, Saini SK, Prinja S. 2014. Impact of Janani Shishu Suraksha Karyakram on out-of-pocket expenditure among urban slum dwellers in northern India. *Indian Pediatrics.* 51(6):475–477.

Tripathy JP, Jagnoor J, Prasad BM, Ivers R. 2018. Cost of injury care in India: cross-sectional analysis of National Sample Survey 2014. *Injury Prevention.* 24(2):116-122. doi: 10.1136/injuryprev-2017-042318. Epub 2017 Jul 19.

Tripathy JP, Prasad BM, Shewade HD, et al. 2016. Cost of hospitalisation for non-communicable diseases in India: are we pro-poor?. *Tropical Medicine and International Health*. 21(8):1019‐1028. doi:10.1111/tmi.12732

Tripathy JP, Shewade HD, Mishra S, Kumar AMV, Harries AD. 2017. Cost of hospitalization for childbirth in India: how equitable it is in the post-NRHM era? *BMC Research Notes.* 10(1):409.

Tripathy JP, Prasad BM. 2018. Cost of diabetic care in India: An inequitable picture. *Diabetes Metab Syndrome.* 12(3):251-255.

Verma VR, Kumar P, Dash U. 2021. Assessing the household economic burden of non-communicable diseases in India: evidence from repeated cross-sectional surveys. *BMC Public Health.* 21(1):881. doi: 10.1186/s12889-021-10828-3.

Wang H, Torres LV, Travis P. 2018. Financial protection analysis in eight countries in the WHO South-East Asia Region. *Bulletin of the World Health Organization.* 96(9):610-620E. doi: 10.2471/BLT.18.209858. Epub 2018 Jul 17.

World Health Organization (WHO) & World Bank. 2019. Global Monitoring Report on Financial Protection in Health 2019.

Wu DC, Sheel V, Gupta P, Essue BM, Luong L, Jha P. 2020. Impact of cigarette tax increase on health and financing outcomes in four Indian states. *Gates Open Research.* doi: 10.12688/gatesopenres.13127.1.

Yadav J, Menon G, Agarwal A, John D. 2021. Burden of injuries and its associated hospitalization expenditure in India. *International Journal of Injury Control and Safety Promotion.* 28(2):153-161. doi: 10.1080/17457300.2021.1879163.

Yadav J, Allarakha S, Menon GR, John D, Nair S. 2021. Socioeconomic Impact of Hospitalization Expenditure for Treatment of Noncommunicable Diseases in India: A Repeated Cross-Sectional Analysis of National Sample Survey Data, 2004 to 2018. *Value in Health Regional Issues.* 24:199-213. doi: 10.1016/j.vhri.2020.12.010.

Yadav J, John D, Allarakha S, Menon GR. 2021. Rising healthcare expenditure on tuberculosis in India: Can India achieve the End-Tb goal? *Tropical Medicine and International Health.* doi: 10.1111/tmi.13648.

Yadav J, John D, Menon G. 2019. Out of pocket expenditure on tuberculosis in India: Do households face hardship financing?. *Indian Journal of Tuberculosis*. 66(4):448‐460. doi:10.1016/j.ijtb.2019.02.016

Yadav J, Menon GR, John D. 2021. Disease-Specific Out-of-Pocket Payments, Catastrophic Health Expenditure and Impoverishment Effects in India: An Analysis of National Health Survey Data. *Applied Health Economics and Health Policy.* doi: 10.1007/s40258-021-00641-9

# Household Sampling

This section describes the complex multi-stage clustered sampling design adopted for selecting the households for our survey. The multiple hierarchical levels of clusters of the household sample were as follows:

1. District
2. Block
3. Villages in rural areas or enumeration blocks in urban centers—referred to as primary sampling units or PSUs
4. Household

**Selection of Districts**

The first level of the household sample was the district. Odisha has 30 districts that are classified under three administrative regions, Regional Development Clusters (RDC), each containing 10 districts. The three RDCs in the state are North, South and Central. Each RDC has differences in demographic and socio-economic characteristics such as poverty levels, proportion of tribal population, proportion of urbanization and industrialization. Therefore, districts were stratified by RDCs.

The district selection was done using a multi-step approach that aimed to represent all sections of the socio-economic, demographic, and geographical characteristics of the state. The following variables were considered for assignment of a “development” index to each district:

1. Population in the district
2. Percentage of urban population
3. Percentage of tribal population
4. District Gross Domestic Product (DGDP)
5. Level of poverty
6. Level of female literacy

Principal Component Analysis (PCA) was conducted for all 30 districts of the state with the above-mentioned six variables. The first Principal Component (PC1) was used to divide the 30 districts into tertiles as this component explained ~50 percent of the total variability in the data. The districts were then stratified by the three RDCs and tertiles within their respective stratum (RDC) (Table A.2).

Two districts from each RDC – one with a high tertile and the other with a low tertile – were selected through with-replacement random sampling. The randomization was through a computer-generated algorithm, and the process was repeated until we obtained a unique list of two districts with different tertiles under each RDC. With-replacement random sampling was chosen over without-replacement, as the former ensures that each district has an equal probability of being sampled.

From this process, we selected the following six districts:

- North RDC: Jharsuguda and Keonjhar
- South RDC: Kalahandi and Rayagada
- Central RDC: Balasore and Khorda

**Table A.2 Tertile Positions of the 30 districts in Odisha based on Principal Component Analysis**

Our sample yielded districts with a range of different characteristics. Among the sampled districts (highlighted in Table A.2), Jharsuguda, Kendujhar, Kalahandi, and Rayagada have a high concentration of Scheduled Tribe (ST) population; except Jharsuguda, all other districts are predominantly rural; Jharsuguda and Kendujhar have mining industries while the other districts are largely agricultural; Kendujhar, Balasore, Kalahandi, and Rayagada have high poverty levels. This distribution of characteristics reflect the diversities of the state to a large extent.

**Selection of Blocks**

The second level of clusters for the sample was the ‘block,’ which is the sub-district administrative unit in India.^[[1]](#footnote-1)^ A total of 30 blocks were selected from the six sampled districts. To select blocks, the following methods were used:

1. First, to determine the number of blocks to be chosen from each district, the method of Proportionate Stratified Sampling (PSS) was used —the district being the stratum and the proportion determined by the population of the district. Based on this, districts with bigger population sizes contributed a greater number of blocks to the sample than districts with smaller populations.
2. From each district, the blocks were sampled using Probability Proportional to Size (PPS).35 This ensured that the larger the population size of the block, the greater the chances of its inclusion in the sample.

First, the cumulative population of all blocks in each district was calculated. Then the total population of the district was divided by the sample size of blocks required from that district (Table A.1). This number generated the Sampling Interval (SI). A computer-generated Random Start (RS) number between zero and the Sampling Interval was used. The population boundaries of the block that contained the RS number was selected as the first block (Populationblock>RS). For selecting the subsequent blocks, the RS number was added to multiples of the Sampling Interval, the second block was selected by Populationblock>(RS+1SI), the third block by Populationblock>(RS+2SI) and so on, till the required number of blocks from each district were sampled.

**Table A.3: Number of blocks required from the sampled districts (based on Proportionate Stratified Sampling), and the blocks sampled (based on Probability Proportional to Size)**

**Selection of Primary Sampling Units**

Primary Sampling Units (PSUs) were the third level of clusters to be sampled. PSUs of each block were stratified into urban and rural (based on census data). ‘Rural’ is defined as census-villages, and ‘Urban’ is defined as census enumeration blocks.

Based on the proportion of rural and urban population in the state, the rural-urban ratio for the sample was determined as 85:15. The sample contained approximately 85 percent rural PSUs and 15 percent urban PSUs. A total of 375 PSUs were sampled from the 30 blocks – 300 villages (rural) and 75 enumeration blocks (urban).

Sampling of PSUs was done using the same methods that we used for sampling blocks. First, Proportionate Stratified Sampling (PSS) was used to determine the number of PSUs that each block would contribute to the sample, calculated based on their population size. 85 percent of this number was the required number of rural PSUs and 15 percent was for urban PSUs. Compared to less populous blocks, more populous blocks contributed a greater number of PSUs to the sample.

Next, once the number of PSUs to be sampled from each block was determined, Probability Proportional to Size (PPS) was used to sample the PSUs in each block. The larger the size of the PSU, the greater the chance of its inclusion in the sample.

The cumulative population of all PSUs in each block was calculated. Then the total population of the block (ΣpopulationPSUs) was divided by the sample size of PSUs required from that block (Table A2.5). This number generated the Sampling Interval (SI). A computer-generated Random Start (RS) number between zero and the SI was used. The population boundaries of the PSU that contained the RS number was selected as the first PSU (PopulationPSU>RS). For selecting the subsequent PSUs, the RS number was added to consecutive multiples of the SI, as in the second PSU was selected by PopulationPSU>(RS+2SI), the third PSU by PopulationPSU>(RS+2SI) and so on, until the required number of PSUs from each block were sampled. The process was repeated till the sample included the required numbers of rural and urban PSUs.

**Table A.4: Number of PSUs needed from the sampled districts (based on Proportionate Stratified Sampling), and the blocks sampled (based on Probability Proportional to Size)**

**A2.2.4 Selection of Households**

To sample households, first, all households in each sampled PSU were listed. From the listing data, the following information about each household in a PSU was collected: (i) Number of households with no event (NE); (ii) Number of households with an outpatient visit in the last 15 days (O); (iii) Number of households with a chronic illness diagnosed by a health provider (C); and (iv) Number of households with hospitalization in the last one year (H).

In addition to the four points of information above, the listing tool also collected information on the ‘preferred provider of the household for outpatient care’ and ‘preferred provider of the household for inpatient care’. While this information was not used for household sampling, this was used for provider sampling (described later in this report).

An absolute precision of 0.007 and a design effect of 2.5 were assigned to arrive at a household sample size of 7500. Considering the rarest event, hospitalization (0.04 or 4.4 percent based on NSSO data), for a sample of 7500 households, the confidence interval (CI) is 95 percent and design effect is 2.5. The number of households sampled from each category were 3000 for no event (NE), and 1500 households each from the outpatient visit (O), chronic illness (C) and hospitalization (H) categories. The oversampling of these latter categories (O, C and H) in comparison to the NE group aims to provide enough precision to stratified estimates of these sub-samples, such as disease-specific expenditures or gender-specific care-seeking frequency. These sample sizes were based on the highest possible number of households that could have these events, as well as the margin of error estimates for variables of interest, for example, expenditure per hospitalization, expenditure per outpatient visit, or percentage referred to chemists. Therefore, the overall margin of error for the estimates of these groups grew even smaller, ranging from 0.000 to 0.001 percent.

The total number of households sampled was 7567. Data was collected about each member of each of these households, so the total number of individuals in the sample was approximately 30,645.

**Table A.5: Sample sizes for households based on screening criteria**

The number of households to be selected from each PSU was fixed at 20. PPS gives us an unequal probability for selection of districts, blocks, and PSUs that results in higher clusters based on their population sizes. However, selection of the same number of elements from unequally sized clusters neutralizes that unequal probability of selection, leading to self-weighting of the sample and equal probability for selection of households. Therefore, 20 HH from each PSU was fixed in order to maintain the integrity of the sample.

To select the 20 households from each PSU, all the households in the PSU were first categorized into the four categories shown above – NE, O, C, H based on the household listing data. These were randomly ordered into MS Excel. The ratio of 2:1:1:1 was followed (based on the sample sizes of 3000 for NE and 1500 each for O, H and C). So, out of the 20 households in each PSU, eight were in NE, and four were from each of the other three categories – O, C, H (not exclusive, some of the households had a combination of these three events).

- Out of the randomly ordered households, the first eight households falling under NE were selected (this included households with no illness in the last 15 days, illness in the last 15 days but none of the other three events, i.e., no chronic illness, no hospitalization, no outpatient visit). This ensured our sample included households that did not access care (foregone care or self-treatment), as well as households that did not have any illness in the last 15 days.
- Out of the randomly ordered households, the first four households that had said “yes” to an outpatient visit in the last 15 days were selected (irrespective of whether they had said “yes” to chronic illness and hospitalization).
- Out of the randomly ordered households, the first four households that had said “yes” to hospitalization in the past one year were selected (again, irrespective of whether they had said “yes” to chronic illness and outpatient visit).
- Out of the randomly ordered households, the first four households that had said “yes” to having been diagnosed with a chronic illness were selected (again, irrespective of whether they had said “yes” to chronic illness and outpatient visit).

There were eight possible categories that came up for households:

1. Households with no event (NE)
2. Households with a diagnosed chronic illness (C)
3. Households with an outpatient visit in the last 15 days (O)
4. Households with an inpatient visit/hospitalization in the past one year (H)
5. Households with an outpatient visit in the last 15 days and a diagnosed chronic illness (C+O)
6. Households with a hospitalization in the past year and a diagnosed chronic illness (C+H)
7. Households with a hospitalization in the past year and an outpatient visit in the last 15 days (O+H)
8. Households with a hospitalization in the past year and an outpatient visit in the last 15 days and a diagnosed chronic illness (C+O+H)

# Weights construction

Sampling weights were computed for the household survey to take into account the multistage sampling strategy and the oversampling of certain types of households. Weights account for three dimensions:

1. Selection of households in each village (PSU/cluster) based on four household characteristics;
2. Selection of each village (PSU/cluster) and block (strata) based on sampling proportionate to the size of the population; and
3. Selection of districts based on a development index.
4. **Selection of households in each village (PSU/cluster) based on four household characteristics**

A listing of each selected village was conducted in which each household was asked whether household members had any of the following:

1. Case of hospitalization in the past year
2. Case of chronic illness
3. Case of outpatient care in the past two weeks
4. None of the above – categorized as a no illness household

In each village, 20 households were selected. For conditions a)-c), 4 households were selected at random to participate in the survey. Among households with no illness, d), 8 households were selected. However, many households had more than one of these conditions, making them eligible to be selected for more than one group. Therefore, we extended the probability of selection to take into account intersections represented by four additional categories:

1. Case of hospitalization and chronic illness
2. Case of outpatient care and chronic illness
3. Case of outpatient care and hospitalization
4. Case of outpatient care, hospitalization and chronic illness

Let each of these conditions be denoted by j. Probability of j or P(j) then is given by:

$$P\left( j \right)=\frac{k_{ji}}{K_{i}} \forall j \in\{1,2,3,4,5,6,7,8\}$$

where {1,2,3,4,$5,6,7,8$} are one of the eight conditions, $k_{ji}$ is the number of households with these conditions that participated in the survey and K_ji_ is the number of HH with condition ‘j’ in cluster ‘i’

1. **Selection of each village (PSU) and block based on sampling proportionate to the size of the population**

Blocks and PSUs were selected based on sampling proportionate to the size of the population. The probability proportionate to size (PPS) based sampling ensures that more populous clusters have a high probability of selection. However, as the same number of individuals are sampled from each cluster, individuals in a larger cluster have a lower probability of selection, which is taken account in step (1). Therefore, in step (2), we calculate just the probability of selection for blocks and PSUs.

Let $P_{P}$be the weighted probability of each cluster being selected – this is calculated based on the number of households in the PSU as a share of all households in the block. Finally, each block in a district has a probability $P_{B}$ of being selected based on the size of the population in the block divided by the population of all blocks in the district. The PPS weights are thus given by:

$$PPS weight=\frac{1}{{(P_{B}*P}_{P} )}$$

Combining these probabilities with the probability from the household conditions, the base weight (BW) for the household survey is given by:

$$BW=\frac{1}{{(P_{B}*P}_{P}* P\left( j \right))}$$

1. **Selection of districts based on the development index of population, female literacy, poverty and gross domestic product, and social groups.**

A development index of population, female literacy, poverty and gross domestic product, and social groups was calculated to categorize districts by development status and select districts based on these strata in addition to geography. We address this selection approach by calibrating our Base Weights to the known population totals of the factors in the development index for the state of Odisha. We use the method of iterative proportional raking to construct these post-stratification weights.

^[[2]](#endnote-1)^ This method minimizes the differences between known population totals and the survey in an iterative manner, raking the distribution in the survey to the known population totals one margin at a time until the differences are minimized across all margins. An adjustment factor ($\pi$) is thus applied to the Base Weights (BW) to produce Raked Weights that represent these population totals.

The final Raked Weight (RW) is thus:

$$RW=BW*\pi$$

We relied predominately on the 2011 Indian census of Odisha to calculate the known population totals.^[[3]](#endnote-2)^ We raked over three margins: 1) the distribution of households by social group (Scheduled Tribe, Scheduled Caste and Other) and rural versus urban residence; 2) the distribution of households with at least one female matriculate (10^th^ grade or higher) by rural and urban residence; 3) the distribution of households with one member above the age of 60; and 4) the share of the population living below the poverty line, based on the population totals from the Reserve Bank of India and the reported possession of a Below Poverty Line card by households in the survey.^[[4]](#endnote-3)^

# Validation of Household Survey with the National Sample Survey

To ensure the validity of the Harvard survey, we compared our results to the results from the National Sample Survey, conducted in the year prior to the Harvard surveys.

**Table A.6: Comparing Basic Household Characteristics**

| **Indicator** | **Harvard Survey** | **NSS 75** |
| --- | --- | --- |
| Number of households | 7,550 | 4,264 |
| Share scheduled tribe | 22% | 26% |
| Share scheduled caste | 17% | 22% |
| Share rural | 84% | 83% |
| Number of individuals | 30,654 | 19,392 |
| Share female | 50% | 49% |
| Share married | 56% | 51% |
| Share under 5 | 8% | 6% |
| Share under 18 | 31% | 31% |
| Share over 60 | 11% | 8% |
| Share ailing in last 15 days | 11% | 10% |
| Share of ailing in the last 15 days that did not seek treatment* | 10% | 21% |
| Share of individuals using care in last 15 days* | 10% | 7% |
| Share using care in the last 15 days in private sector | 30% | 29% |
| Median spend per visit in the last 15 days | 400 | 390 |
| Share of spending on drugs† | 57% | 39% |
| Share with hospitalization last 365 days | 4% | 4% |
| Share of hospitalizations in the private sector | 23% | 23% |
| Median spending per hospitalization | 5000 | 4000 |
| Share of spending on drugs | 32% | 25% |

Notes: SC/ST and share over 60 based on census and thus correct; *Harvard survey is slightly higher because of time of year and no adjustments to seasonality have been implemented; †Higher because of inclusion of chemist visits – NSSO did not consider that. How “Self-treatment” calculated: Harvard survey: No treatment / self-treatment based on selecting either: self-treated, did not do anything or A friend or family went and bought medicines for me in response to “After you fell ill, what did [NAME] do? (other options: Sought treatment/medical advice from a provider only, both self-treated and sought treatment; A friend or family member consulted the provider (chemist, doctor, ANM/ASHA) on my behalf and got medicines for me. NSS 75: No to: whether treatment taken on medical advice

**Table A.7: Comparing Catastrophic Health Expenditure**

|  | **Harvard** | **NSSO 75** |
| --- | --- | --- |
| **CHE (10%), visit-based** | 24% | 24% |
| **CHE (25%), visit-based** | 12% | 13% |
| **Households with any OOP**  **(visit-based)** | 38% | 38% |
| **Average monthly health USD (visit-based)** | 1049 | 489 |

**Table A.8 Comparing care in the last 15 days overall, by location and gender**

|  | **Overall** | | **Urban** | | **Rural** | | **Male** | | **Female** | |
| --- | --- | --- | --- | --- | --- | --- | --- | --- | --- | --- |
|  | **Harvard** | **NSSO** | **Harvard** | **NSSO** | **Harvard** | **NSSO** | **Harvard** | **NSSO** | **Harvard** | **NSSO** |
| **Ailing** | 11% | 10% | 10% | 12% | 12% | 9% | 11% | 9% | 12% | 10% |
| **Where: Public Facility** | 48% | 43% | 53% | 47% | 47% | 42% | 50% | 45% | 45% | 41% |
| **Where: Private Facility** | 30% | 29% | 28% | 29% | 31% | 29% | 27% | 29% | 33% | 29% |
| **Median Spend: Public** | 470 | 320 | 400 | 300 | 500 | 350 | 400 | 320 | 500 | 345 |
| **Median Spend: Private** | 500 | 500 | 700 | 500 | 500 | 500 | 500 | 500 | 600 | 500 |
| **No treatment/ Self- treatment*** | 10% | 21% | 8% | 18% | 10% | 21% | 8% | 17% | 11% | 24% |
| **Why: not sick enough** | 85% | 85% | 96% | 71% | 82% | 88% | 77% | 86% | 92% | 84% |
| **Why: too expensive** | 9% | 7% | 4% | 10% | 10% | 6% | 13% | 5% | 6% | 8% |

**Table A.9: Comparing care in the last 15 days by Social Group**

|  | **Scheduled Tribe** | | **Scheduled Caste** | | **Scheduled Tribe** | |
| --- | --- | --- | --- | --- | --- | --- |
|  | **Harvard** | **NSSO** | **Harvard** | **NSSO** | **Harvard** | **NSSO** |
| **Ailing** | 9% | 8% | 12% | 8% | 13% | 11% |
| **Where: Public Facility** | 41% | 45% | 48% | 43% | 49% | 42% |
| **Where: Private Facility** | 33% | 21% | 32% | 25% | 29% | 33% |
| **Median Spend: Public** | 250 | 300 | 485 | 345 | 500 | 370 |
| **Median Spend: Private** | 340 | 350 | 600 | 500 | 600 | 535 |
| **No treatment/ Self- treatment*** | 14% | 22% | 8% | 25% | 9% | 19% |
| **Why: not sick enough** | 77% | 93% | 89% | 88% | 87% | 80% |
| **Why: too expensive** | 14% | 1% | 7% | 12% | 8% | 7% |

**Table A.10: Comparing hospitalizations overall, by location and by gender**

|  | **Overall** | | **Urban** | | **Rural** | | **Male** | | **Female** | |
| --- | --- | --- | --- | --- | --- | --- | --- | --- | --- | --- |
|  | **NSSO** | **Harvard** | **NSSO** | **Harvard** | **NSSO** | **Harvard** | **NSSO** | **Harvard** | **NSSO** | **Harvard** |
|  | 4% | 4% | 4% | 4% | 4% | 5% | 3% | 3% | 5% | 6% |
| **Private share** | 23% | 23% | 28% | 42% | 23% | 20% | 28% | 30% | 20% | 20% |
| **Median Total OOP** | 5000 | 4000 | 5400 | 5300 | 5000 | 3960 | 6500 | 5170 | 5000 | 3750 |
| **Drug OOP share** | 32% | 25% | 34% | 23% | 31% | 25% | 34% | 25% | 30% | 25% |
| **% Any reimb-urse-ment** | 5% | 2% | 5% | 2% | 4% | 2% | 6% | 3% | 4% | 2% |

**Table A.11: Comparing hospitalizations by Social Group**

|  | **Scheduled Tribe** | | **Scheduled Caste** | | **Other** | |
| --- | --- | --- | --- | --- | --- | --- |
|  | **NSSO** | **Harvard** | **NSSO** | **Harvard** | **NSSO** | **Harvard** |
| **Any hospitalization** | 2% | 4% | 5% | 5% | 4% | 5% |
| **Private share** | 14% | 8% | 18% | 18% | 27% | 30% |
| **Median Total OOP** | 2000 | 2550 | 4000 | 3420 | 6400 | 5330 |
| **Drug OOP share** | 21% | 24% | 34% | 26% | 34% | 25% |
| **% Any reimbursement** | 1% | 2% | 6% | 2% | 5% | 2% |

# Mapping of facility types

| **Table A.12: Facility name and the level categorized to** | | |
| --- | --- | --- |
| **#** | **Facility name** | **Level** |
| 1. | Medical College and tertiary hospital | Public Hospital |
| 2. | District Hospital/Municipal Hospitals (urban) | Public Hospital |
| 3. | Sub-District Hospital | Public Hospital |
| 4. | Community Health Center/First Referral Unit/Rural Hospital (CHC/FRU) | Public Hospital |
| 5. | Urban Health Center/Urban PHC/Urban Health Post | Public Primary |
| 6. | Primary Health Center (PHC) | Public Primary |
| 7. | Sub-Center/Health and Wellness Center (SC/HWC) | Public Primary |
| 8. | ASHA (Community Health Worker) | Public Primary |
| 9. | Anganwadi Center | Public Primary |
| 10. | AYUSH Hospital/Clinic | Ayush |
| 11. | Government pharmacy/Niramaya or Jan Aushadhi drug stores | Public Chemist |
| 12. | Mobile Medical Unit | Public Primary |
| 13. | Health Camp | Private Primary |
| 14. | NGO/Trust/Charitable Hospital  NGO/ | Private Hospital |
| 15. | Private hospital (tertiary or secondary) | Private Hospital |
| 16. | Nursing homes (secondary) | Private Hospital |
| 17. | Maternity homes | Private Hospital |
| 18. | Dispensary | Private Primary |
| 19. | Chemist shops | Private Chemist |
| 20. | Diagnostic Laboratories | Private Other |
| 21. | Registered doctors (solo-practice) | Private Primary |
| 22. | AYUSH doctors (solo practice) | Private Ayush |
| 23. | Bengali doctor, or other names | Private Other Non-provider |
| 24. | Traditional healer | Private Other Non-provider |
| 25. | Other stores (E.g. Grocery stores) | Private Other Non-provider |

# Mapping of reason for pursuing care

**Table A.13: Cause response option and the reason categorized to**

| **Reason** | **Cause response options** |
| --- | --- |
| Acute Respiratory | - Acute upper respiratory infections (cold, runny nose, sore throat with cough, allergic colds included) (36) |
| Cancer* | - Cancers (known or suspected by a physician) and occurrence of any growing painless lump in the body (13) |
| Child Birth | - Pregnancy with complications before or during labour (abortion, ectopic pregnancy, abortion, hypertension, complications during labour) (49) - Complications in mother after birth of child (50) - Childbirth – Caesarean/ normal/ any other (for both live birth and stillbirth) (88) - Ante-Natal care (61) - Post-Natal Care (62) |
| Diabetes* | - Diabetes (16) |
| Diarrhea | - Diarrheas/ dysentery/ increased frequency of stools with or without blood and mucus in stools (11) |
| Fever | - Fever with loss of consciousness or altered consciousness (1) - Fever with rash/ eruptive lesions (2) - Fever due to diphtheria, whooping cough (3) - All other fevers (Includes malaria, typhoid and fevers of unknown origin, all specific fevers that do not have a confirmed diagnosis) (4) |
| Heart Disease* | - Hypertension (34) - Heart disease: Chest pain, breathlessness (35) |
| Injury | - Accidental injury, road traffic accidents and falls (52) - Accidental drowning and submersion (53) - Burns and corrosions (54) - Poisoning (like food, water) (55) - Intentional self-harm (56) - Assault (57) - Contact with venomous/harm-causing animals and plants (58) |
| Local Pain or Weakness* | - Weakness in limb muscles and difficulty in movements (24) - Stroke/ hemiplegia/ sudden onset weakness or loss of speech in half of body (25) - Pain the pelvic region/reproductive tract infection/ Pain in male genital area(47) - Pain in abdomen: Gastric and peptic ulcers/ acid reflux/ acute abdomen (40) - Back or body aches (45), |
| Other | - All other response options – refer to household survey tool. |
| Skin Infection* | - Skin infection (boil, abscess, itching) and other skin disease (43) |

Note: Reasons were grouped according to whether they had similar epidemiological characteristics and were a commonly cited response option. *Initially mapped to this category and then remapped to other because of a small number of responses for the purpose of analysis.

# Decomposition analysis results

**Table A.14: Explained variation by covariate and covariate group**

|  | OOP per outpatient visit | OOP per hospitalization | Any OOP in the household | OOP / Consumption Expenditure | CHE 10% | Distress Financing |
| --- | --- | --- | --- | --- | --- | --- |
| Age | 0.6% | 2.6% | - | - | - | - |
|  | (0.2% - 1.3%) | (0.8%-5.4%) |  |  |  |  |
| Cause | 1.5% | 11.9% | 20.7% | 11.0% | 7.3% | 10.2% |
|  | (0.5%-3.0%) | (6.8%-17.8%) | (19.0%-22.8%) | (9.6%-12.5%) | (6.1%-8.6%) | (7.7%-13.1%) |
| Chronic Diagnosis | 2.6% | 2.1% | - | - | - | - |
|  | (1.0%-4.8%) | (0.4%-4.5%) |  |  |  |  |
| Days Missed | 5.2% | - | 7.0% | 9.3% | 11.1% | 6.0% |
|  | (2.3%-9.0%) |  | (6.3%-8.0%) | (8.3%-10.7%) | (9.0%-13.4%) | (3.9%-9.1%) |
| HH Member Older than 60 | - | - | 0.1% | 0.1% | 0.1% | 0.1% |
|  |  |  | (0.1%-0.2% | (0.1%-0.3%) | (0.0%-0.3%) | (0.1%-0.3%) |
| HH Member with Chronic Diagnosis | - | - | 3.4% | 2.7% | 1.9% | 3.1% |
|  |  |  | (2.8%-3.9%) | (2.2%-3.2%) | (1.4%-2.5%) | (1.9%-4.5%) |
| Level | 11.8% | - | - | - | - | - |
|  | (7.9%-16.2%) |  |  |  |  |  |
| Number of Drugs | 30.6% | - | 13.9% | 16.4% | 17.2% | 3.1% |
|  | (21.5%-39.7%) |  | (12.7%-15.2%) | (14.6%-18.0%) | (14.5%-20.2%) | (2.2%-4.2%) |
| Number of Hospitalizations | - | - | 11.4% | 10.4% | 8.3% | 50.1% |
|  |  |  | (9.7%-12.6%) | (9.3%-11.5%) | (6.6%-10.1%) | (45.2%-54.9%) |
| Number of Outpatient Visits | - | - | 19.7% | 21.7% | 23.1% | 5.5% |
|  |  |  | (18.4%-21.0%) | (20.5%-22.8%) | (21.0%-25.7%) | (3.7%-7.7%) |
| Poor Self-reported Health | - | 1.6% | - | - | - | - |
|  |  | (0.1%-4.2%) |  |  |  |  |
| Primary Education Only | 0.3% | 2.5% | - | - | - | - |
|  | (0.1%-0.8%) | (0.4%-5.9%) |  |  |  |  |
| Private Drugs | 38.7% | - | 17.5% | 18.9% | 19.1% | 2.6% |
|  | (30.1%-47.1%) |  | (16.2%-18.7%) | (17.6%-20.5%) | (16.9%-21.1%) | (1.8%-3.5%) |
| Private Facility | 1.3% | 43.9% | - | - | - | - |
|  | (0.8%-2.2%) | (34.5%-52.5%) |  |  |  |  |
| Private Hospitalization | - | - | 2.0% | 3.5% | 4.8% | 11.0% |
|  |  |  | (1.7%-2.3%) | (2.8%-4.1%) | (3.7%-5.9%) | (7.8%-14.7%) |
| Private Outpatient Care | - | - | 3.3% | 4.8% | 5.7% | 3.4% |
|  |  |  | (3.0%-3.6%) | (4.1%-5.5%) | (4.5%-7.1%) | (1.9%-4.9%) |
| Report Use of Insurance | - | 6.8% | - | - | - | - |
|  |  | (1.5%-13.8%) |  |  |  |  |
| Reported Use of Insurance for Hosp. | - | - | 0.2% | 0.2% | 0.2% | 1.0% |
|  |  |  | (0.2%-0.4%) | (0.1%-0.3%) | (0.1%-0.4%) | (0.6%-1.6%) |
| Rural/Urban | 6.1% | 17.8% | 0.1% | 0.2% | 0.3% | 0.9% |
|  | (3.4%-9.4%) | (11.3%-24.8%) | (0.0%-0.1%) | (0.1%-0.3%) | (0.1%-0.5%) | (0.5%-1.3%) |
| Share of HH with Primary Education Only | - | - | 0.1% | 0.1% | 0.2% | 0.1% |
|  |  |  | (0.0%-0.1%) | (0.0%-0.2%) | (0.1%-0.4%) | (0.1%-0.3%) |
| Social Group | 0.6% | 1.4% | 0.3% | 0.3% | 0.3% | 0.6% |
|  | (0.1%-1.9% | (0.3%-3.4%) | (0.1%-0.5%) | (0.1%-0.5%) | (0.1%-0.5%) | (0.3%-1.1%) |
| Wealth Quintile | 0.7% | 9.4% | 0.2% | 0.3% | 0.6% | 2.3% |
|  | (0.2%-1.7%) | (5.1%-14.5%) | (0.1%-0.4%) | (0.2%-0.4%) | (0.3%-0.9%) | (1.5%-3.2%) |
| Healthcare Determinants | 82.4% | 50.7% | 68.0% | 76.0% | 78.3% | 76.7% |
|  | (76.1%-87.6%) | (41.1%-60.0%) | (66.4%-69.6%) | (74.1%-77.9%) | (76.0%-80.7%) | (72.0%-80.1%) |
| Number of Drugs + Private Drugs | 69.3% | - | 31.4% | 35.3% | 36.3% | 5.7% |
|  | (61.3%-76.2%) |  | (29.6%-33.2%) | (32.5%-37.5%) | (32.7%-40.1%) | (4.2%-7.5%) |
| Health Determinants | 9.3% | 15.6% | 31.2% | 23.1% | 20.3% | 19.3% |
|  | (5.5%-14.0%) | (9.5%-22.2%) | (29.5%-33.0%) | (21.2%-24.9%) | (17.8%-22.5%) | (16.2%-23.0%) |
| Social Determinants | 8.3% | 33.7% | 0.8% | 1.0% | 1.4% | 4.0% |
|  | (5.3%-12.1%) | (25.6%-42.9%) | (0.5%-1.1%) | (0.7%-1.2%) | (0.9%-2.0%) | (3.0%-5.3%) |
| R-Squared | .394 | .255 | .752 | .749 | .534 | .312 |

# Essential medicine list stocks

**Table A.15: Essential medicines in stock by facility type**

|  | **Public secondary & tertiary facilities** | **Public primary facilities** | **Private chemists** |
| --- | --- | --- | --- |
| Average share of essential medicines in stock | 62% | 31% | 48% |

**Table A.16: Drugs surveyed for availability on the day of the survey**

| **Product** |
| --- |
| AMITRIPTYLINE CHLORDIAZEPOXIDE |
| AMOXYCILLIN |
| AMPICILLIN |
| AMLODIPINE |
| VITAMIN C |
| ASPIRIN |
| ATENOLOL |
| ATORVASTATIN |
| CALCIUM GLUCONATE |
| CARBAMAZEPINE |
| CETRIZINE |
| CHLOROQUIN |
| CHLORPHENIRAMINE MALEATE |
| CIPROFLOXACIN |
| COTRIMOXAZOLE |
| DEXAMETHASONE |
| DICLOFENAC |
| DICYCLOMINE |
| DOMPERIDONE |
| DOXYCYCLINE |
| ENALAPRIL |
| ERYTHROMYCIN |
| FERROUS SULPHATE AND FOLIC ACID |
| FOLIC ACID |
| FRUSEMIDE |
| GENTAMICIN |
| GLIMEPIRIDE |
| GLIPIZIDE |
| IBUPROFEN |
| ISOSORBIDE DINITRATE |
| METFORMIN |
| METRONIDAZOLE |
| NIFEDIPINE |
| ORS PACKET |
| PARACETAMOL |
| PENICILLIN G POTASSIUM |
| PHENOBARBITONE |
| PHENYTOIN SODIUM |
| PREDNISOLONE |
| RANITIDINE |
| RABIES VACCINE |
| SODIUM BI-CARBONATE |
| SALBUTAMOL |
| SODIUM VALPROATE |
| THEOPHYLLINE AND ETOFYLLINE |
| TRIHEXYPHENIDYL |
| LEVO THYROXIN |
| VITAMIN B COMPLEX |
| ZINC |
| NORADRENALINE |
| ARTESUNATE |
| LIGNOCAINE |
| MAGNESIUM SULPHATE |
| OXYTOCIN |
| SURGICAL SPIRIT |
| TETANUS TOXOID |
| TINIDAZOLE |
| VITAMIN A |
| METHYLCOBALAMINE |
| NORFLOXACIN |
| OMEPRAZOLE |

# Opening hours of different facility types

**Table A.17: Average hours open per day by facility type**

|  | **Public secondary & tertiary facilities** | **Public primary facilities** | **Private chemists clustered around public facilities** |
| --- | --- | --- | --- |
| Average hours open per day | 9 | 7 | 11 |

# OOP spending by wealth quintile

**Table A.18: Average OOP expenditure by wealth quintile**

| **Wealth Quintile** | **OOP health spending (rupees)** | **Drug OOP health spending (rupees)** | **Drug OOP share of total OOP** | **Hospital OOP health spending (rupees)** | **Hospital OOP share of total OOP** |
| --- | --- | --- | --- | --- | --- |
| 1 | 6,457 | 4,471 | 71% | 1,145 | 20% |
| 2 | 8,140 | 4,601 | 65% | 1,782 | 24% |
| 3 | 11,616 | 5,881 | 64% | 2,976 | 21% |
| 4 | 12,941 | 7,432 | 67% | 3,464 | 21% |
| 5 | 18,547 | 9,122 | 58% | 5,918 | 30% |

# CHE rates by sector & wealth quintile

**Table A.19: CHE rates and utilization by sector & wealth quintile**

| **Outpatient visits** | | | | | | |
| --- | --- | --- | --- | --- | --- | --- |
| Wealth Quintile | CHE (10%) | CHE (10%) in public sector | Public sector share of visits | CHE (10%) in the private sector | Public share of all CHE cases in wealth quintile | Share of all CHE cases |
| 1 | 36% | 37% | 42% | 45% | 42% | 22% |
| 2 | 26% | 29% | 49% | 33% | 54% | 19% |
| 3 | 30% | 29% | 52% | 39% | 51% | 25% |
| 4 | 25% | 26% | 49% | 26% | 52% | 21% |
| 5 | 15% | 14% | 45% | 23% | 40% | 13% |
| **Hospitalizations** | | | | | | |
| Wealth Quintile | CHE (10%) | CHE (10%) in public sector | Public sector share of visits | CHE (10%) in the private sector | Public share of all CHE cases in wealth quintile | Share of all CHE cases |
| 1 | 26% | 22% | 87% | 49% | 76% | 14% |
| 2 | 24% | 19% | 83% | 46% | 68% | 15% |
| 3 | 32% | 24% | 83% | 73% | 62% | 25% |
| 4 | 32% | 19% | 74% | 68% | 44% | 24% |
| 5 | 22% | 13% | 63% | 39% | 36% | 22% |

1. On an average, a district comprises of 10 blocks. The average population of a district is 1,000,000, and of a block is 100,000. There is wide variation among districts and blocks across the country as well as within Odisha. [↑](#footnote-ref-1)
2. # References

   . Kolenikov S. 2014. Calibrating survey data using iterative proportional fitting. *The Stata Journal.* Volume 14, Number 1, pp. 22=59. [↑](#endnote-ref-1)
3. . Census of India. 2011. Available at: https://censusindia.gov.in/2011census/population_enumeration.html (Accessed December 2020). [↑](#endnote-ref-2)
4. . Reserve Bank of India.2015. Number and Percentage of Population Below Poverty Line. Available at: https://web.archive.org/web/20170602074949/https://www.rbi.org.in/SCRIPTs/PublicationsView.aspx?id=16603 (Accessed April 2021). [↑](#endnote-ref-3)
